# ALC: automated reduction of rule-based models

**DOI:** 10.1186/1752-0509-2-91

**Published:** 2008-10-31

**Authors:** Markus Koschorreck, Ernst Dieter Gilles

**Affiliations:** 1 Max Planck Institute for Dynamics of Complex Technical Systems, Sandtorstr. 1, 39106 Magdeburg, Germany

## Abstract

**Background:**

Combinatorial complexity is a challenging problem for the modeling of cellular signal transduction since the association of a few proteins can give rise to an enormous amount of feasible protein complexes. The layer-based approach is an approximative, but accurate method for the mathematical modeling of signaling systems with inherent combinatorial complexity. The number of variables in the simulation equations is highly reduced and the resulting dynamic models show a pronounced modularity. Layer-based modeling allows for the modeling of systems not accessible previously.

**Results:**

ALC (Automated Layer Construction) is a computer program that highly simplifies the building of reduced modular models, according to the layer-based approach. The model is defined using a simple but powerful rule-based syntax that supports the concepts of modularity and macrostates. ALC performs consistency checks on the model definition and provides the model output in different formats (C MEX, MATLAB, *Mathematica *and SBML) as ready-to-run simulation files. ALC also provides additional documentation files that simplify the publication or presentation of the models. The tool can be used offline or via a form on the ALC website.

**Conclusion:**

ALC allows for a simple rule-based generation of layer-based reduced models. The model files are given in different formats as ready-to-run simulation files.

## Background

### Combinatorial complexity

The association of a few proteins can result in an enormous amount of possible protein complexes [1,2]. This phenomenon is referred to as combinatorial complexity. A general observation is that there are four possible sources of combinatorial complexity in signaling systems that can also occur in combination with each other:

• Binding events at scaffold proteins with more than one binding site

• Modification (e.g. phosphorylation) of proteins with several sites

• Oligomerization (as a special case: dimerization)

• Chain formation

In a simplified form, we previously used the insulin signaling system as a demonstration object for combinatorial complexity [3]. The complexity (145,156,469 possible species) results from a combination of the first three items listed above. Many other signaling systems also show combinatorial complexity [1]. As outlined below, one ODE (ordinary differential equation) is required for the balance of each possible complex. This leads to enormous problems in the modeling of systems with inherent combinatorial complexity.

### Structure of the introduction

In the following sections, we introduce general modeling concepts which are characterized by significant differences in their ability to cope with combinatorial complexity. We outline the conventional approach to model biochemical systems, discuss its limitations and review recent techniques that allow for the modeling of systems with inherent combinatorial complexity. A very promising approach is that of layer-based modeling [3], for which we present ALC (Automated Layer Construction), a tool for rule-based automated modeling. The main motivation of this comprehensive introduction is to provide simplified but accurate information regarding layer-based modeling for those who are not familiar with the method. More involved discussions and several examples can be found in our previous publication introducing layer-based modeling [3]. Readers who are already familiar with rules and layer-based modeling should read the "general concepts and definitions" section but may skip the remainder of the introduction and directly proceed to the "implementation" chapter where we introduce the software tool ALC.

### General concepts and definitions

In this section, we introduce the notation for the variables and their dependencies that we use later on. Proceeding in such a formal way may not be necessary for conventional modeling, however, introducing the layer-based approach and ALC benefits from a precise terminology.

#### Modules

Systems can often be decomposed into self-contained subsystems that interact with other subsystems in a well-defined way. Such subsystems are called modules. The process of defining the modules and their connections is called modularization.

#### Molecule definitions

The molecule definition introduced here is not equivalent to the strict chemical understanding of molecules where all atoms have to be linked by covalent bonds. A molecule definition represents the class of possible modifications of a protein (or another molecule). It consists of the molecule name that is optionally followed by the successive definition of all sites (if there are any). A site definition consists of a comma-separated sequence of all possible modifications of this site, enclosed by curly brackets. As an example for a molecule definition, R{0, L}{0, P} defines the molecule *R*. The possible modifications (or configurations) of *R *are '0' and 'L' at the first site as well as '0' and 'P' at the second site. Note that we allow for the consideration of a bound ligand (e.g. *L*) as a site configuration of the receptor molecule. There can also be molecules that exist in only one configuration. The modification of them results in new molecules.

#### Species and concentrations

Species correspond to a specific configuration of a molecule and can be considered as instances of the corresponding molecule definitions. Notations for species consist of the molecule name followed by the comma-separated sequence of site configurations within one pair of squared brackets. As an example, the species *R*[*L*, *P*] is a specified configuration of the molecule *R*. The ligand *L *is bound to the first site, the second site is phosphorylated. The species *R*[0, 0] defines the unphosphorylated receptor without ligand. If a molecule *A *can occur in just one state (i.e. the molecule definition is A), this single species is also denoted as *A*. For the sake of simplicity, we use the same notation for species and their concentrations.

#### Macroscopic variables

In exact model reduction [4,5] and layer-based modeling [3] there exist variables that correspond to sums of species of a corresponding conventional model. We call these variables macroscopic variables (or lumped states). Note that species can be macroscopic variables.

#### Microscopic species

All species that are not macroscopic variables are microscopic species (or microstates). Microscopic species correspond to a distinct modification of a molecule and do not represent a pool of chemical molecules or complexes with common properties. Microscopic species are species that could also occur in a conventional model. Therefore, in the conventional modeling approach, microscopic species are equivalent to species.

#### Macrostates and patterns

Macrostates describe macroscopic variables, e.g. degrees of phosphorylation or occupancy which correspond to sums of species. Their notation is analogous to that for species, with the sole difference being that the modification 'X' at a specific site indicates that all distinct modifications at this site are included. Therefore, a site modification 'X' of a molecule indicates the sum of all possible modifications of this site. Macrostates in rules are interpreted as patterns. Each species of the class defined by the pattern occurs in a separate reaction when the rule is evaluated. Therefore, patterns do not represent sums of species (as macrostates do) but each of the corresponding species individually (see section "rule-based modeling"). Like species, macrostates can also be considered as instances of the corresponding molecule definitions. Note that not all macroscopic variables are macrostates. The hallmark of macrostates and patterns is that they must contain at least one site modification 'X'.

As an example, the macrostate *ERB*[*L*, *X*, *X*] defines the sum of all species of the molecule *ERB *that have a bound ligand at the first site, regardless of the state of the other sites.

Note that the term macrostate is used with a slightly different meaning in other contributions [4,5], where it represents what we call a macroscopic variable (or macroscopic species). We reserve the term macrostate for sums of species that are defined by at least one site modification 'X'. The so defined macrostates are also macrostates or mesoscopic states as defined by Borisov et al. [4] and Conzelmann et al. [5].

#### Complexes

A complex is represented by a list of comma-separated species within curly brackets where the binding partner is indicated at each occupied binding site. Indices have to be used if it is required to achieve uniqueness.

As an example, consider the binding of a molecule *R *with *n *sites and a molecule *S *with *k *sites. The complex of *R *and *S *is {*R*[*m*_1_,..,*S*,...,*m*_*n*_], *S*[*m*_1_,...,*R*,...,*m*_*k*_]}, where *m*_*i *_denotes the modification at the site position *i*. If we also consider another molecule *T *that has *q *sites, the complex of *R*, *S *and *T*, where *S *and *T *bind to *R *is denoted as {*R*[*m*_1_,..,*S*,...,*T*,...,*m*_*n*_], *S*[*m*_1_,...,*R*,...,*m*_*k*_], *T*[*m*_1_,...,*R*,...,*m*_*q*_]}. This definition of complex notation is very general but cumbersome in many cases.

We introduce a simplified notation that will be used from now on and is supported by ALC. In many cases, this notation is less cumbersome and more intuitive than the general one. Consider the case that the molecules *S *and *T *both have only one site which is a binding site for *R*. The complex of *R*, *S *and *T *then is *R*[*m*_1_,..,*S*,...,*T*,...,*m*_*n*_] and can be treated as a species of the molecule *R*. If *S *and *T *have more sites, the configuration of *S *and *T *in the complex with *R *can be indicated by introducing additional site modifications of the corresponding site on *R*. If two of the associating molecules have several sites, the class of possible complexes can be defined by a new molecule definition which consists of a new molecule name and the sequence of site definitions of the corresponding molecule definitions for the monomers. We exemplify this for the general case where *R *has a binding site for *S *and *n *other sites, while *S *has a binding site for *R *and *k *other sites. A species representing a complex of *R *and *S *is $RS[m_{1}^{R},...m_{n}^{R},m_{1}^{S},...m_{k}^{S}]$, where the superscripts indicate the origin of the sites. Dimerization can be treated analogously.

Due to the modular structure of layer-based models, this simplified notation is usually more convenient than the general one, even if the signaling network is highly branched and contains many scaffold proteins.

#### Summary

Modules are self-contained subsystems of a system that interact with other subsystems in a well-defined way. Species and macrostates are instances of molecule definitions which in turn define classes of species. Species can be microscopic species that correspond to a distinct modification of a chemical molecule or a complex. Species can also be macroscopic variables that correspond to sums of microscopic species. Macrostates are macroscopic variables that correspond to sums of species and whose hallmark is that they must contain at least one site modification 'X'. Patterns occurring in rules have the same notation as macrostates and define classes of species. Each species of such a class occurs in a separate reaction when the rule is evaluated. Following the simple notation of complexes, they are treated as species of one of the participating molecules or as species of newly defined molecules.

### Reactions and kinetics

#### Mass action kinetics

If chemical systems are modeled at the molecular level, mass action kinetics are often a good description of the chemical processes [6] and are therefore frequently used. We use a representation of reaction equations which includes the dynamic information by adding the kinetic parameters of the reaction. In a reaction such as

(1) *aA *+ *bB*... ⇋ *cC *+ *dD*...    *k*1*   k*1*d*

*A*, *B*, *C *and *D *are the participating species and *a*, *b*, *c *and *d *are their stoichiometric coefficients. The kinetic parameters of the forward and backward reactions are *k*1 and *k*1*d*, respectively. According to the law of mass action [6], the reaction rate *r *for this reaction (Equation 1) is given as

(2) *r *= *k*1·*A*^*a*^·*B*^*b*^... - *k*1*d*·*C*^*c*^·*D*^*d*^...

where *A*, *B*, *C *and *D *are the concentrations of the corresponding species. Taking the stoichiometric coefficients as the exponents in the rate law is only justified if the system is homogeneous and if the mixture is ideal [6,7]. We will always assume that these assumptions are fulfilled.

Reactions may be irreversible which can be indicated by a reaction parameter that equals zero or a reaction symbol that indicates the direction of the reaction ('→' or '←' instead of '⇋'). In the latter case, only one kinetic parameter has to be given.

#### Generalized mass action kinetics

In the classical law of mass action for ideal mixtures, *k*1 and *k*1*d *are constants [6] which only depend on the temperature and on the pressure [7]. In the generalized law of mass action presented in [6], the constants *k*1 and *k*1*d *are multiplied by a common positive nonlinear function (that may also depend on concentrations of species). We use a generalized law of mass action that is more general where both reaction parameters may be general nonlinear functions.

#### Enzyme kinetics

Michaelis-Menten kinetics are a simplified formal description of enzyme activity that assumes an irreversible conversion of the substrate *S *to the product *P *[6,7]. A reaction following Michaelis-Menten kinetics can be formulated according to the generalized law of mass action

(3) $$\begin{array}{cc} S\to P & \frac{r_{max}}{K_{M}+S} \end{array}$$

which leads to the reaction rate

(4) $$r=r_{max}\cdot \frac{S}{K_{M}+S}.$$

Note that the power of *S *that is 'missing' in the numerator of the parameter expression in Equation 3 is re-introduced by building the rate law according to the generalized law of mass action (Equation 4). There exist several extension of Michaelis-Menten kinetics that can also be formulated by the generalized law of mass action [6].

## Conventional modeling and its limitations

The conventional (mechanistic) modeling approach for biochemical networks is to define each chemical reaction by a reaction equation. A rate law has to be assigned for each reaction and one ODE is required for the balance of each microscopic species [6]. This approach is frequently applied in systems biology [8-17].

Conventional modeling is very powerful for small reaction systems however its limitations become clearly visible when considering systems with inherent combinatorial complexity. For these systems, the numbers of necessary reactions and ODEs are very high [2]. As an example, 1.5·10^8 ^ODEs would be necessary to get a conventional model of the insulin signaling system [3]. Therefore, the modeling of systems with inherent combinatorial complexity is very difficult, or even impossible using the conventional modeling approach. A frequently used solution of this problem is to simplify the combinatorial variety by focusing on small subsets of the possible reactions and complexes. This method can work well and is often applied [8-15], but there are also cases in which the resulting models show poor accuracy when compared to a model which considers all possible complexes and reactions.

The reason for these differences in approximation quality is that it is very difficult to decide which reactions and complexes can be neglected and which cannot. Apparently well-founded assumptions may result in large approximation errors [5]. The structure of the reduced model (i.e. the decision of which species and reactions can be neglected; the assumed temporal order of the processes) depends on the parameter values of the reaction network [18]. However, these parameters are often not known and can only be estimated after the set-up of the model equations.

A few systematic approaches for the modeling of systems with inherent combinatorial complexity exist. In the following sections, first of all we outline the exact modeling techniques and then give an introduction to layer-based modeling; a recent approximative technique.

## Rule-based modeling

A single molecular event, e.g the binding of an effector, is often described by a long list of reactions. Large subsets of these reactions or even all reactions are usually parameterized by the same kinetic constants. Such subsets can be represented using generalized reactions with patterns instead of species [2,19,20]. These generalized reactions are called rules and each of them represents a class of reactions that are parameterized by the same kinetic constants. In many cases, a relatively short list of rules is sufficient to describe the reactions of a signaling system. Software tools exist [20-26] which automatically generate the simulation files from a rule-based model definition.

We exemplify the usage of rules using a simple example. The following reactions represent ligand binding to a receptor that has an additional site which can be phosphorylated.

(5) $$\begin{array}{ccc} R[0,0]+L⇋R[L,0] & k1 & k1d\\ R[0,P]+L⇋R[L,P] & k1 & k1d \end{array}$$

These reactions can be expressed by a single rule containing patterns on both sides.

(6) *R*[0, *X*] + *L *⇋ *R*[*L*, *X*] *k*1 *k*1*d*

Sites with a modification 'X' at the same position on both sides of the reaction equation correspond to each other. When rules are evaluated, the species in each reaction have the same modification at this site on both sides of the reaction equation.

Rules can also be parameterized by generalized mass actions kinetics in the same way as reactions. We give an example for enzyme kinetics of rules in the ALC user guide (Additional file 1).

Altogether, rules highly simplify the model representation and the generation of the model equations. A major drawback of rule-based modeling is that the resulting model may be very large as one ODE is required for each feasible species. The concept of rules however, is very powerful and is also used in stochastic simulation, exact model reduction and layer-based modeling, all of which are introduced below.

## Stochastic simulation

Chemical processes at the molecular level show a stochastic behavior which is described by the chemical master equation [27]. Approaches directly reflecting the stochasticity describe integer populations of distinct species and assign to each reaction a probability for its occurrence in a certain time interval. Software tools exist that allow for the stochastic simulation of a rule-based model [21-26].

There are two major approaches for the stochastic simulation; namely on-the-fly and generate-first [28]. The generate-first approach uses rules to define the total reaction network of the system which is used for the stochastic simulation. The on-the-fly approach allows for the reduction of the model size. During the simulation, all complexes are only balanced if they are populated. The reactions of the complexes are also only generated if their occurrence is possible, i.e. if the complexes that act as reactants are populated. The advantage of the reduced model size comes at the price of a high computational effort if many simulation runs are necessary, e.g. for parameter estimation.

We do not discuss the stochastic approaches in detail and focus on deterministic modeling using ODEs. This approach describes chemical processes well, if the number of molecules and the volume are large enough [27].

## Exact model reduction

Exact model reduction aims to achieve a macroscopic description of biochemical systems. This is motivated by the view that domains (functional components of proteins) and not species are the fundamental elements of signal transduction [29]. State variables in exactly reduced models are macroscopic quantities, such as levels of occupancy or degrees of phosphorylation that represent the state of the domains. These macroscopic quantities are preserved in exactly reduced models. Information about all single species is usually not of interest and is no longer explicitly contained in the models. In the last few years, two exact techniques for the reduction of deterministic models have been developed [4,5].

Borisov et al. proposed the first exact approach to reduce the enormous amount of possible model equations [4]. The starting point is a rule-based model definition from which the reduced model is directly generated. However, the method only considers modularity within a single molecule with many sites. A major deficiency of this method is that the modularity of the entire reaction network cannot be considered. Therefore, this method is very valuable for describing complex formation at one large scaffold protein, however, it cannot be applied to many real signaling systems.

Conzelmann et al. [5] extended and generalized the approach by proposing an exact domain-oriented lumping technique which can be applied to a large class of systems. However, for the application of this method it is necessary to evaluate the rule-based model definition and to use a conventional ODE model as the starting point. The conventional ODE model (that can be very large) is subjected to a state space transformation which results in the exactly reduced model if the unobservable states are omitted. The formal reduction method presented by Conzelmann et al. [5] has recently been extended and improved [30,31]. The modified approach facilitates the generation of the exactly reduced model equations and does not require the former generation of a complete conventional model any more.

As in the approach of Borisov et al. [4], the performance of domain-oriented lumping [5,31] strongly depends on the interactions in the network. Enormous reduction is possible in some cases. In other cases no reduction is possible [3]. In many cases, the resulting reduced models still consist of too many ODEs for efficient simulation or analysis.

## Layer-based modeling

Layer-based modeling allows for a macroscopic and highly reduced description of signaling systems [3]. A very convenient feature of layer-based modeling is that the reduced model can be directly obtained, which means in particular that the preceding generation of a conventional model is not necessary. The number of necessary ODEs in layer-based models is dramatically reduced. As an example, modeling of the insulin signaling system is possible with only 214 ODEs, instead of the 1.5·10^8 ^needed when using conventional mechanistic modeling [3].

A graph of all processes and their interactions serves to define the modules that are called layers. The different layers are connected in a highly standardized way. They either exchange signals that correspond to macroscopic quantities, or they are not connected. No reaction proceeds across layer boundaries, therefore it is possible to model the layers separately from each other once their connections are defined. In special cases, a layer-based model exactly describes the dynamics of macroscopic quantities. In typical biochemical scenarios, it provides an approximative, but highly accurate description.

In this section, we give a detailed introduction to layer-based modeling. We start by introducing the concepts of processes and interactions necessary for the building of interaction graphs, which define the modularity of layer-based models. Afterwards we describe how the layers can be modeled and how their connections are defined. At the end of the introduction we discuss the mathematical background and the approximation quality of layer-based modeling.

### Processes

In combinatorial reaction networks, the same molecular event often occurs in many different reactions. The high number of reactions results from the high amount of different species that participate in the reactions. All reactions that describe the same molecular event define the corresponding process. As an example, all reactions that change a certain level of occupancy or degree of phosphorylation belong to the same process. Note that the inverse molecular event also belongs to the same process (e.g. the process effector binding also contains all reactions describing effector dissociation).

The reactions of a process may either be parameterized by the same or by different kinetic constants depending on the presence of process interactions.

### Interactions

Interactions between processes result in different parameter values for different reactions of the same process. This means that the reactions of a process that is influenced by other processes are not parameterized by the same kinetic constants. If the reaction parameters of a process depend on a modification that is performed by another process, these two processes interact with each other. If reaction parameters of two processes do not depend on the other process, these two processes do not interact.

There exist three structurally different types of interactions between processes. They can interact via *graded interactions*, *all-or-none interactions *or they do *not interact *[3].

#### Graded interaction

**Figure 1:**
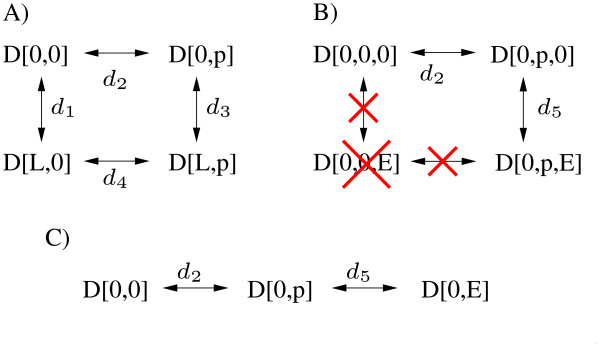
**Types of interactions between processes**. Reactions are indicated by arrows. The reaction rate for each reaction is denoted as *d*_*i *_and parameterized by *k*_*i *_and *k*_-*i*_. A) Visualization of a graded interaction between the processes of *L *binding and *D *phosphorylation. The same scheme can be taken to demonstrate non-interacting processes. For non-interacting processes the parameter restrictions *k*_1 _= *k*_3_, *k*_-1 _= *k*_-3_, *k*_2 _= *k*_4 _and *k*_-2 _= *k*_-4 _hold. B) Visualization of an all-or-none interaction between the processes of binding site phosphorylation and *E *binding. *E *can only bind to phosphorylated sites, while the dephosphorylation of *D *is only possible in the absence of a bound effector. C) The cyclic reaction scheme degenerates to a reaction chain, as the species *D*[0, 0, *E*] does not exist. In this case, the notation can be simplified by describing binding site phosphorylation and *E *binding as modifications of the same site.

Processes that undergo graded interactions can influence each other in arbitrary ways. A typical case is the binding of a ligand that influences the kinetic parameters for receptor phosphorylation (Figure 1A). Graded interactions can be unidirectional or mutual. Therefore, receptor phosphorylation may, but does not have to influence the kinetic parameters for ligand binding. If each process is defined by two reactions, these four reactions can be arranged as a reaction cycle. If the processes are defined by more reactions, there are also more cycles.

#### All-or-none interaction

All-or-none interactions are a limiting case of graded interactions, where both processes can only occur under certain preconditions, provided by the other process. A typical case is the interaction of the processes of binding site phosphorylation and effector binding (Figure 1B). The effector can only bind if the binding site is phosphorylated. Additionally, the dephosphorylation of the binding site is only possible if the site is not occupied. A hallmark of all-or-none interactions is the degeneration of reaction cycles to reaction chains (Figure 1C).

Phosphorylation and effector binding are the most common examples of processes that undergo an all-or-none interaction. Such an interaction is also possible for other pairs of processes, e.g ligand binding and receptor dimerization (dimerization occurs only if the ligand is bound, ligand dissociation is only possible for monomers), or receptor dimerization and receptor phosphorylation (phosphorylation is only possible if the receptor is dimerized, dissociation is only possible if the receptor is unphosphorylated). To simplify the discussion, and without loss of generality, we will always assume that the processes that undergo all-or-none interactions are phosphorylations and effector bindings.

#### No interaction

The third type of interaction, also a limiting case of graded interactions, is that the processes do not interact. Kinetic parameters of each process are not influenced by the other process (Figure 1A).

### The interaction graph and layers

**Figure 2:**
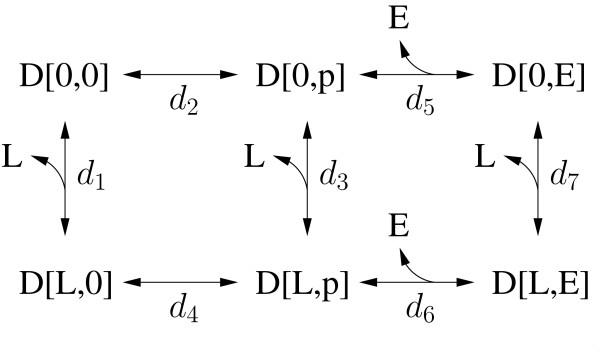
**A conventional mechanistic representation of the example system**. A receptor *D *has two sites and is defined as D{0, L}{0, p, E}. The first site is a binding site for the ligand *L*, the second is a binding site for the effector *E *that has to be phosphorylated before *E *can bind. Reactions are indicated by arrows. The reaction rate for each reaction is denoted as *d*_*i*_.

**Figure 3:**
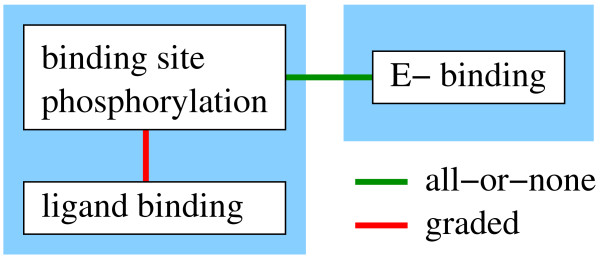
**Interaction graph and modularization of the small example system**. All processes (white boxes) that are coupled by graded interactions (red lines) are described in the same layer (blue boxes). Processes of different layers are only connected by all-or-none interactions (green lines) or do not interact.

The interaction graph is a systematic visualization of the processes and their interactions. After identifying the processes and their interactions, building the interaction graph is the second step in layer-based modeling. In the interaction graph, the processes (represented by boxes) are connected by lines indicating their interactions. All-or-none interactions are represented by green lines, graded interactions are represented by red lines. A small example system (Figure 2) that is discussed below contains the following processes: ligand binding, binding site phosphorylation and effector binding. The interaction graph of this system is shown in Figure 3.

The third step in layer-based modeling consists of determining the modularity of the model from the interaction graph. All processes that are directly or indirectly connected by graded interactions form a module that is called layer. The set of these processes can be directly obtained from the interaction graph, as all processes that are connected by red lines (representing graded interactions) are to be described within the same layer. Note that layers may only contain a single process, and that there may be many layers. The connectivity of the layers is defined by all-or-none interactions between the processes of the different layers. After the definition of the connections, the layers are modeled independently from each other, as if all processes belonging to other layers do not exist.

In contrast to other modularization techniques [32-35] where the modularity is deduced from the entire model, layers can be modeled independently from each other once their connections are defined. This may give the impression that the user defines the modularity, however, the modularity is uniquely defined by the interaction pattern of the processes. Once the modeler has defined which processes interact in which way, they have only one way of influencing the modularity, which is to merge two or more layers into a larger one.

### Mass flow and signal flow

In this subsection, we discuss the characteristics of mass flows and signal flows, and their occurrence in layer-based modeling. This helps in the understanding of basic principles of layer-based modeling, as the flows within layers and between layers are qualitatively different.

#### Mass flow

Each reaction defines a transition between species whose quantity is given by the reaction rate. We refer to these transitions as mass flows. Mass flows occur within layers and do not cross layer boundaries. Note that fluxes without spatial direction (e.g. within a compartment) are also considered as mass flows.

#### Signal flow

Signal flows define information transfers. In contrast to mass flow, signal flow is not associated to reactions. In layer-based modeling, signal flows occur between layers and can be represented by macroscopic variables, usually sums of species with phosphorylated binding sites *x*_*i *_and corresponding sums of species with occupied binding sites *x*_*i*_*b*. These signals are exchanged between layers containing processes that interact via all-or-none interactions. This will be discussed in detail below.

#### Flows and layers

Layers are either only connected by signal flows or are not connected at all. No mass flows cross layer boundaries as no reactions transport substance from one layer to the other. Within layers there are mass flows defined by reaction equations and the corresponding rates as in conventional modeling. The connections between layers show retroactivity [34] because the signal flow is bidirectional. However, changes in a layer (e.g. the introduction of an additional regulatory site) that do not affect the notation of *x*_*i *_and *x*_*i*_*b *are usually not followed by changes in another layer. Therefore, the layers can be modeled separately from each other as long as the notations of the signal flows between the layers are defined.

### Modeling of layers

In the fourth step of layer-based modeling, the signal flows between the layers are defined and the layers are modeled separately from each other. Modeling within layers shows remarkable similarities to conventional modeling, however, there are differences that mainly result from the presence of all-or-none interactions between processes of different layers.

#### Phosphorylation of binding sites

All-or-none interactions between binding site phosphorylations and the corresponding effector bindings bring much of the reduction potential of layer-based modeling. An all-or-none interaction implies that a phosphorylated binding site remains phosphorylated while the effector is bound, and that the effector binds only to phosphorylated binding sites. The phosphorylation of a binding site is often described in a different layer than the layer in which effector binding to the phosphorylated binding site is described. However, if there are graded interactions that connect the binding site phosphorylation and effector binding processes indirectly, these two processes have to be described in the same layer. It is obvious that the phosphorylation of binding sites to which effector binding is described in another layer has to be treated differently than the phosphorylation of regulatory sites or the phosphorylation of binding sites to which the effectors bind in the same layer.

To indicate the phosphorylation of a binding site to which the effector binds in another layer, the site notation (uppercase) 'P' is used. The phosphorylation of other sites can be indicated by other notations, e.g. (lowercase) 'p'. Species with a site modification (uppercase) 'P' comprise microscopic species with a phosphorylated but unoccupied binding site as well as microscopic species with a phosphorylated and occupied binding site.

Effector binding to phosphorylated binding sites in other layers does not directly change the concentration of species with a site modification 'P'. Therefore, layers only exchange signal flows and not mass flows across layer boundaries. The signal flows between the layers are used to guarantee that only unoccupied binding sites can be dephosphorylated (see below).

#### Reactions

The processes of each layer are described by reactions as if there were no other layers. Note that this also holds for the phosphorylation of binding sites to which effector binding is described in another layer. The sole difference is that the phosphorylation of binding sites to which the effectors bind in other layers has to be indicated by a site modification (uppercase) 'P'.

Effectors can only bind to a subset of phosphorylated binding sites, namely those that are not occupied. Effector binding to such a binding site in another layer is performed by introducing a new species that represents the sum of all microscopic species with phosphorylated but unoccupied binding sites. This new species acts as a binding partner for the effector and is defined by an algebraic equation. It is defined as the difference of the sum of species with phosphorylated binding sites *x*_*i *_and the corresponding sum of species with occupied binding sites *x*_*i*_*b*. These sums define the signal flows between the layers and are discussed in the next paragraph.

#### Layer connections

For the realization of all-or-none interactions between two processes of different layers, signal flows have to be exchanged between the layers. These signal flows are typically defined by the sums of species with phosphorylated binding sites *x*_*i *_and the corresponding sums of species with occupied binding sites *x*_*i*_*b*. Note that the signal flows *x*_*i *_and *x*_*i*_*b *often correspond to experimental readouts. These signals are used to compute correction terms assuring that only unoccupied binding sites are dephosphorylated (see below, e.g. Equation 7). Their differences define the concentrations of binding partners for effectors whose binding site phosphorylations are described in different layers.

#### Rates and ODEs

Reaction rates are assigned to reactions as in conventional modeling. In most cases generalized mass action kinetics are used, however, the layer-based approach also allows for other kinetics. The sole exception from this analogy is the dephosphorylation of binding sites with a site modification 'P'. Without a special treatment of these dephosphorylation reactions, generalized mass action kinetics result in an overestimation of the rates for the dephosphorylation, as occupied binding sites are also dephosphorylated. Remember that the site modification 'P' represents the phosphorylation of a binding site without distinguishing if an effector is bound or not. Therefore, a correction term *c*_*i *_has to be introduced for the dephosphorylation of each binding site with a modification 'P'.

(7) $$c_{i}=\frac{x_{i}-x_{i}b}{x_{i}}$$

It represents the fraction of species with phosphorylated binding sites that are not occupied. The need for these correction terms results from the loss of information and the corresponding reduction of the model size that is due to the modularization of the network. The correction terms always have the same structure, however, different *x*_*i *_and the corresponding *x*_*i*_*b *are taken for each binding site.

The rate for each dephosphorylation reaction of a binding site with a site modification 'P' is multiplied by the appropriate *c*_*i *_which guarantees that only unoccupied binding sites are dephosphorylated.

All dephosphorylation rates for a specific site with a modification 'P' are multiplied by the same *c*_*i*_. The implicit assumption behind this is that all species with this phosphorylated binding site (that all count to the same *x*_*i*_) have the same fraction of unoccupied phosphorylated binding sites. This is the core of the approximation and the reduction and results from the rapid equilibrium assumptions that are discussed in the mathematical background.

The ODEs are generated as they would be in conventional modeling. Note that no ODE is necessary for the binding partners in binding reactions that are defined by algebraic equations as differences of *x*_*i *_and *x*_*i*_*b*. The sums *x*_*i *_and *x*_*i*_*b *are also defined by algebraic equations.

#### A small example system

In a small example system that has already been intensively discussed [3], it is assumed that a receptor *R *has two sites. The first is a binding site for the ligand *L*, the second is a binding site for the effector *E*. This binding site has to be phosphorylated before *E *can bind. A conventional representation of this system is shown in Figure 2. To simplify the discussion, the receptor is denoted as *D *in the conventional *detailed *model and is denoted as *R *in the layer-based *reduced *model.

The processes of ligand binding and autophosphorylation perform a graded interaction (Figure 1A). In this special case, ligand binding stimulates the autophosphorylation, whereas the autophosphorylation does not influence ligand binding. The processes of autophosphorylation and effector binding undergo an all-or-none interaction (Figure 1B). Note that another reaction chain that is analogous to the one in Figure 1C and includes microscopic receptor species with a bound *L *exists.

**Figure 4:**
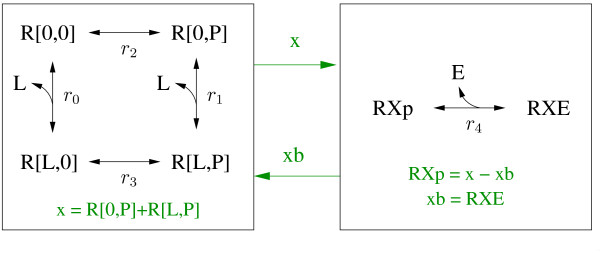
**Layer-based reaction network of the example system**. The receptor layer (left) describes the processes of ligand binding and receptor phosphorylation by two reactions each. The effector layer (right) describes the effector binding process by one reaction. The signals between the layers are the sum of species with a phosphorylated binding site *x *and the sum of species with an occupied binding site *xb *(which in this case is only the species RXE). Black arrows indicate reactions, green arrows indicate signal flows.

The interaction graph of this system is shown in Figure 3. As all processes that are coupled by graded interactions belong to the same layer, the reduced model of the example system consists of two layers. The receptor layer describes the processes of ligand binding and receptor phosphorylation, the effector layer describes the process of effector binding. The reaction scheme of the reduced model is shown in Figure 4. The model equations of the receptor layer are

(8a) $$\begin{array}{l} r_{0}=k1\cdot L\cdot R[0,0]-k1d\cdot R[L,0]\\ r_{1}=k1\cdot L\cdot R[0,P]-k1d\cdot R[L,P]\\ r_{2}=k2\cdot R[0,0]-k2d\cdot ((x=xb)/x)\cdot R[0,P]\\ r_{3}=k3\cdot R[L,0]-k3d\cdot ((x-xb)/x)\cdot R[L,P] \end{array}$$

(8b) ddtR[0,P]=−r1+r2ddtR[L,0]=r0−r3ddtR[L,P]=r1+r3R[0,0]=totR−(R[0,P]+R[L,0]+R[L,P])L=10

where *totR *is the total concentration of the receptor. In this example, the concentration of the (extracellular) ligand *L *is set to the constant value 10.

Consider the correction term in the expression for dephosphorylation in the rates *r*_2 _and *r*_3_. The microscopic receptor species *D*[0, *p*] and *D*[*L*, *p*] (see Figure 2) are approximated from the species *R*[0, *P*] and *R*[*L*, *P*] using the correction term *c *= (*x *- *xb*)/*x *to guarantee that only unoccupied binding sites are dephosphorylated.

(9) $$\begin{array}{c} D[0,P]\approx \frac{x-xb}{x}\cdot R[0,P]\\ D[L,P]\approx \frac{x-xb}{x}\cdot R[L,P] \end{array}$$

The model equations of the effector layer are

(10a) *r*_4 _= *k*4·*RXp*·*E *- *k*4*d*·*RXE*

(10b) ddtRXE=r4RXp=x−xbE=totE−RXE

where *totE *is the total concentration of the effector and *RXp *is a species that represents the sum of all microscopic receptor species with phosphorylated but unoccupied binding sites. The analogy to conventional modeling can be clearly seen in this layer as the reaction rate, the ODE and the conservation relation for the effector could also occur in a conventional model describing the association of two species. The signal flow between the layers is defined by *x *and *xb*, where x is the sum of species with phosphorylated binding sites and *xb *is the sum of species with occupied binding sites.

(11) $$\begin{array}{c} x=R[X,P]=R[0,P]+R[L,P]\\ xb=RXE \end{array}$$

Equations 8 and 10 show that the layers can be modeled independently from each other as long as the connections between the layers (Equation 11) are defined.

Note that the letter 'X' only indicates a macrostate if it is a site configuration. In the species *RXE *and *RXp*, the 'X' is a part of the molecule name. Therefore, *RXE *and *RXp *are not macrostates. However, *RXE *represents the sum of all microscopic species where *E *is bound to the receptor, and *RXp *represents the sum of all microscopic species where the binding site is phosphorylated and not occupied. Therefore, both are macroscopic species.

#### Advanced strategies

Using the descriptions that have been provided above, it is possible to model a large class of systems by applying the step by step procedure below. Here we introduce advanced strategies that allow for an additional reduction of layer-based models. All strategies are exemplified by model definitions for ALC resulting in executable models (Additional file 2).

##### Equivalent binding sites

If *n *binding sites on a molecule are exactly equivalent and do not influence each other (and if this molecule is not an oligomer that can dissociate) it is sufficient to model only one binding site. Note that this holds only if the initial conditions of the degrees of phosphorylation are also identical.

In this case, the *x*_*i *_for this binding site results from multiplying the sum of species with phosphorylated binding sites by *n*, e.g. *x*_*i *_= *n*·*R*[*P*]. For linear phosphorylation or dephosphorylation kinetics, the rate law is equivalent to the case where each site is modeled separately. If the factor *n *is considered in the definition of *x*_*i *_one needs not to consider this factor in any reaction. For nonlinear phosphorylation or dephosphorylation kinetics, e.g. Michaelis-Menten kinetics, *n *times the concentration of the corresponding species has to be used as the substrate concentration in the rate laws.

(12) $$r_{tot}=\frac{r_{max}\cdot n\cdot R[0]}{n\cdot R[0]+K_{M}}$$

In addition, the total rate *r*_*tot *_has to be divided by *n *because *r*_*tot *_is the sum of the rates for all *n *sites and we only consider one of them.

(13) $$r=\frac{r_{tot}}{n}$$

The rate *r *describes the kinetics of the phosphorylation of *R*[0] to *R*[*P*]. According to the generalized law of mass action, the reaction and its parameterization is given as

(14) $$\begin{array}{cc} R[0]\to R[P] & \frac{r_{max}}{n\cdot R[0]+K_{M}}. \end{array}$$

If it is desired to have a model output representing the sum of all phosphorylated binding sites (for both linear and nonlinear kinetics), the factor *n *has to be considered and the output is *n*·*R*[*P*].

Equivalent sites that are not binding sites and that do not influence each other can be treated in an analogous way. The reduction potential resulting from equivalent binding sites can be exploited to exactly reduce the model of insulin signaling with 214 ODEs [3] to 51 ODEs (Additional file 3).

##### One effector binding to different binding sites

This is highly related to the case above, but now *m *different binding sites (not necessarily belonging to the same molecule) are equivalent with respect to effector binding and may have different phosphorylation characteristics. A common *x*_*i *_for all *m *binding sites can be used. This *x*_*i *_equals the sum of all sums of species with phosphorylated binding sites for the effector, $x_{i}=x_{i_{1}}+\ldots +x_{i_{m}}$. Note that species with *j *phosphorylated binding sites for the effector count *j *times in *x*_*i*_. A frequent application of this is effector binding to a receptor *R*_1 _that can dimerize to *R*_2_. If the binding of *E *only depends on binding site phosphorylation, a common *x*_*i *_can be used in the layer connections.

(15) xR=R1[P]+R2[P,X]+R2[X,P]xRb=E[R]RXp=xR−xRb

*RXp *represents the sum of all phosphorylated but unoccupied binding sites on the dimers and monomers of the receptor. The Binding of *E*[0] to *RXp *leads to *E*[*R*].

The correction term *c*_*R *_for the dephosphorylation of all these binding sites is the same and equals *c*_*R *_= (*x*_*R *_- *x*_*R*_*b*)/*x*_*R*_. This corresponds to the assumption that the same fraction *c*_*R *_of phosphorylated binding sites is occupied for all those sites with a site modification 'P'.

This strategy also prevents the danger of negative concentrations in the effector layers that may occur if e.g. receptor dimerization is faster than effector dissociation and *R*_1_[*P*] rapidly 'vanishes' due to dimerization. In an example (where the actual advanced strategy is not applied) *x*_*R*1 _= *R*[*P*, *X*,...] is the sum of monomers with phosphorylated binding sites and the associated *x*_*R*1_*b *represents all effectors that are bound to receptor monomers. Let the system be in a state where a large fraction of the binding sites is occupied, i.e. *x*_*R*1 _- *x*_*R*1_*b *≈ 0. Assume that an external stimulus induces the rapid dimerization of the receptor which rapidly lowers the value of *x*_*R*1 _because the dimers are not included in *x*_*R*1_. Note that the dimerization has no direct influence on *x*_*R*1_*b*. Therefore, it is possible that the concentration of the species *R*1*Xp *= *x*_*R*1 _- *x*_*R*1_*b *representing the concentration of the sum of unoccupied binding sites of the monomer becomes smaller than zero. As mentioned above, this problem can and should be avoided by using a common *x*_*i *_for corresponding sites on the monomers and dimers.

##### Additional signals between layers

Additional signals between layers are allowed as long as no graded interactions are introduced by them. In a typical example, the processes of receptor activation and receptor phosphorylation are described in the receptor layer, whereas the binding and phosphorylation of effectors is described in different layers. The phosphorylation of effectors that are bound to receptors is performed by activated receptors. It is important that one specific activated receptor does not selectively phosphorylate effector molecules that are bound to it. This would introduce a graded interaction between the processes of receptor activation and effector phosphorylation and therefore destroy the modular structure of the model. Instead of this, it is advantageous to assume that the pool of activated receptors acts as an enzyme and phosphorylates receptor-bound effectors. In this case, the signal representing receptor activity can be transferred to the effector layers as an additional signal.

In general, the additional coupling between processes of different layers must occur in the form of a mean-field assumption. This means that the rate of an event in a downstream layer (e.g. effector phosphorylation) is proportional to the average value of some property computed from the upstream layer (e.g. receptor activation).

#### Step by step procedure: layer-based modeling

This step by step procedure can be used for efficient and standardized layer-based modeling.

1. Identify all processes and their interactions.

2. Draw the interaction graph.

3. Deduce from the interaction graph how many layers are in the model and which processes form part of which layer. All processes that are coupled by graded interactions are part of the same layer. Processes of different layers are coupled by all-or-none interactions or do not interact.

4. Model each layer separately as if the processes of all other layers do not exist.

(a) Define rules and reactions, as in conventional modeling. The site modification (uppercase) 'P' is reserved for indicating the phosphorylation of binding sites to which effectors bind in other layers. A different notation (e.g. lowercase 'p') has to be used to indicate the phosphorylation of other sites.

(b) Define all sums of species with phosphorylated binding sites *x*_*i *_and all sums of species with occupied binding sites *x*_*i*_*b*. This only has to be done for binding sites with a site modification 'P'. Note that *x*_*i*_*b *has the same notation as the corresponding *x*_*i*_, followed by (lowercase) 'b'.

(c) Use algebraic equations to define the concentrations of the species that act as binding partners in effector binding to binding sites whose phosphorylation is described in other layers. These species are defined as differences of *x*_*i *_and *x*_*i*_*b*.

(d) Assign a rate to each reaction, as in conventional modeling. A special case is the dephosphorylation of binding sites with a site modification (uppercase) 'P'. The rate expressions for these dephosphorylation reactions have to be multiplied by the appropriate correction term *c*_*i *_= (*x*_*i *_- *x*_*i*_*b*)/*x*_*i*_. Using the correction terms *c*_*i *_guarantees that the dephosphorylation is only possible for unoccupied binding sites.

(e) Conservation relations can be used to replace one ODE for each conserved moiety (e.g. a protein that is not degraded or synthesized).

(f) Use reactions and rates, as in conventional modeling, to build the ODEs for all species that are not defined by an algebraic equation.

(g) Assign initial conditions guaranteeing *x*_*i *_> 0 (prevents division by zero) and *x*_*i *_≥ *x*_*i*_*b *∀ *i *(prevents negative concentrations of binding partners).

#### Mathematical background

In the following subsection, we give an overview about the mathematical background of layer-based modeling. We do not recapitulate the detailed discussions provided elsewhere [3], but give the main results and some additional descriptive interpretations. Understanding the mathematical background is not necessary for layer-based modeling. Therefore, this subsection may be skipped if the reader is not interested in mathematical details.

We start the discussion by describing the rapid equilibrium assumption, which is an important part of the theoretical basis for the reduction potential of layer-based modeling.

#### Rapid equilibrium assumption

If in a reaction network

(16) $$\begin{array}{c} \ldots ⇋S\overset{r_{i}}{\underset{}{⇋}}P⇋\ldots \\ r_{i}=k_{i}\cdot S-k_{-i}\cdot P \end{array}$$

the parameters *ki *and *k*_-*i *_are much larger than the parameters of the remaining reactions, the quotient *P*/*S *will, initially after a short period of time, approximately equal the equilibrium constant [6].

(17) $$\frac{P}{S}\approx \frac{k_{i}}{k_{-i}}=K_{i}$$

This reaction is almost in equilibrium though a nonzero flux *r*_*i *_goes through it. Equation 17 can be used to replace one ODE of the system. The resulting reduced model provides a good approximation of the dynamics after a short initial time span (or for all times if the initial conditions already fulfill Equation 17) [6].

#### Diagonal reactions

**Figure 5:**
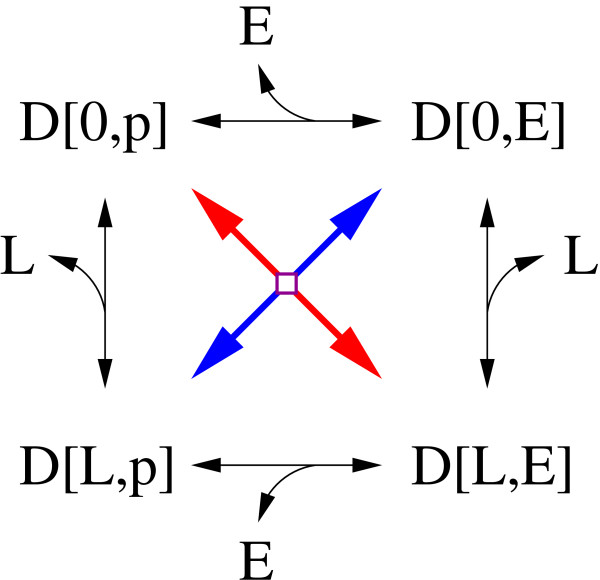
**Equilibrium assumption and diagonal reaction**. The diagonal reaction (Equation 21) whose equilibrium assumption is the basis for model reduction of the example system is symbolized in the middle of the reaction cycle.

Layer-based modeling makes wide use of the rapid equilibrium assumption for processes of different layers that do not interact directly. We demonstrate this for the small example system (Figures 2 and 4) where a reaction cycle is formed by the reactions describing the bindings of *L *and *E *to the phosphorylated receptor (Figure 5). There are four equilibrium conditions for this reaction cycle.

(18) $$\begin{array}{c} \frac{D[0,p]\cdot E}{D[0,E]}=K_{1}\\ \frac{D[L,p]\cdot E}{D[L,E]}=K_{2}\\ \frac{D[0,p]\cdot L}{D[L,p]}=K_{3}\\ \frac{D[0,E]\cdot L}{D[L,E]}=K_{4} \end{array}$$

As the bindings of *E *and *L *do not interact directly (Figure 3), it holds that *K*_1 _= *K*_2 _and *K*_3 _= *K*_4 _which reduces the number of independent equations to three. One of these three independent equations which results from the division of the first equation by the second or from the division of the third equation by the fourth is

(19) $$\frac{D[0,p]}{D[L,p]}=\frac{D[0,E]}{D[L,E]}$$

which implies that the processes of *L *binding and *E *binding are completely independent. However, this is an approximation as there is an indirect interaction between these processes (Figure 3) that results in (dynamic) deviations from Equation 19.

Equation 19 can be rewritten as

(20) *D*[0, *p*]·*D*[*L*, *E*] - *D*[*L*, *p*]·*D*[0, *E*] = 0.

This corresponds to a rapid equilibrium assumption for the reaction

(21) *D*[0, *p*] + *D*[*L*, *E*] ⇋ *D*[*L*, *p*] + *D*[0,*E*]

which is not part of the reaction scheme (Figure 2) and where the equilibrium constant equals one. This reaction fills the diagonals of the square representing the reaction cycle (Figure 5). Therefore, we refer to this kind of fast virtual reaction as a diagonal reaction.

Assuming that the two processes are indeed independent, the equations following from the calculus of probability as discussed by Borisov et al. [4] can be transformed to Equation 20 which leads to a different interpretation of the same equations [3].

As Equation 20 is only one of three equilibrium conditions, assuming equilibrium for the diagonal reaction is a weaker assumption than assuming equilibrium for the whole reaction cycle.

The approximation in layer-based modeling is based on the (implicit) equilibrium assumption for diagonal reactions connecting processes of different layers that do not interact directly. Note that such diagonal reactions can connect more than two processes (for an example see Additional file 4, p.3 in [3]). Each such equilibrium assumption reduces the number of necessary ODEs by one.

#### Non-uniqueness of diagonal reactions

The list of diagonal reactions is not unique. In almost all larger models there are several conditions such as Equation 19, which leads to

(22) *q*_1 _= *q*_2 _= ... = *q*_*n*_

where *q*_*i *_are quotients of microscopic species of the conventional model (see again Additional file 4, p.3 in [3]). Equation 22 provides *n *- 1 independent equations. It can be easily seen that the list of the corresponding diagonal reactions depends on the choice of the *n *- 1 independent equations that result from Equation 22. However, the set of microscopic species connected by the diagonal reactions is unique.

#### Approximation quality

We introduce a measure for the approximation error *g *as the deviation from Equation 20.

(23) *D*[0, *p*]·*D*[*L*, *E*] - *D*[*L*, *p*]·*D*[0, *E*] = *g*

The error *g*(*t*) quantifies the distance of the diagonal reaction (Equation 21, Figure 5) from equilibrium. It follows a first order ODE

(24) $$\begin{array}{cc} \dot{g}+a(t)\cdot g=u(t), & g(0)=g_{0} \end{array}$$

with *a*(*t*) = *k*1·*L *+ *k*1*d *+ *k*4·*E *+ *k*4*d *and *u*(*t*) = *d*_2_·*D*[*L*, *E*] - *d*_4_·*D*[0, *E*], that gives the analytical solution

(25) g(t)=e−A(t)(g0+∫0teA(τ)u(τ)dτ)A(t)=∫0ta(τ)dτ.

Note that *A*(*t*) = *a*·*t *if *a*(*t*) ≡ *a*. For *u*(*t*) ≥ 0, *a*(*t*) > 0 ∀ *t *and *g*_0 _≥ 0, an upper bound for the dynamical error is given by

(26) $$g(t)\leq g_{0}\cdot e^{-a_{min}\cdot t}+\frac{u_{max}}{a_{min}}\cdot \left(1-e^{-a_{max}\cdot t}\right)$$

Where

(27) umax=max⁡0≤τ≤t(u(τ))amin=k1⋅min⁡0≤τ≤t(L(τ))+k1d+k4⋅min⁡0≤τ≤t(E(τ))+k4damax=k1⋅max⁡0≤τ≤t(L(τ))+k1d+k4⋅max⁡0≤τ≤t(E(τ))+k4d.

A very important result from Equation 26 is that the approximation error declines and is bounded because *u*(*t*) is bounded from above and *a*(*t*) is bounded from both below and above. Similar expressions to Equation 26 can be given for *u*(*t*) ≤ 0 and *g*_0 _≤ 0.

There is one error equation (with *g*_*i*_, *a*_*i *_and *u*_*i*_) as Equation 24 for each diagonal reaction. In the most simple case, *a*_*i *_equals the sum of the four kinetic parameters of the reaction cycle. In each case, *a*_*i *_is large if the reaction parameters in the corresponding cycle are large and *u*_*i *_is a weighted sum of external fluxes (that can be positive or negative) entering the reaction cycle. When unbalanced (*u*_*i*_(*t*) ≠ 0), these incoming fluxes can lead to fluxes through the diagonal reaction that lower the approximation quality.

All *u*_*i*_(*t*) vanish in thermodynamic equilibrium or in the absence of graded interactions. Therefore, layer-based modeling provides an *exactly reduced model *if there are no graded interactions (i.e. if all processes are connected by all-or-none interactions or do not interact) or if the layers containing graded interactions are not coupled to the other layers. In both cases, the initial conditions of the corresponding conventional model have to fulfill the equilibrium conditions for the diagonal reactions (e.g. Equation 20). If they do not, the approximation is only *stationarily exact*.

The approximation is also stationarily exact if all fluxes vanish in the stationary case, i.e. if the system reaches thermodynamic equilibrium. Note that a system is able to reach thermodynamic equilibrium if the Wegscheider condition [6] is fulfilled for the entire reaction network and if no species or fluxes are set to a constant value (or follow an externally imposed trajectory). Another possibility for stationary exactness is the presence of a stationary flux distribution, characterized by *u*_*i*_(*t*) ≡ 0 ∀ *i *even if the system does not reach equilibrium.

In all other cases, layer-based modeling is a purely *approximative *method. However, for typical scenarios the approximation error is very low. We showed this for an example system where parameter variations of four orders of magnitude around values from literature were not able to cause a low approximation quality [3].

#### Evaluation of the approximation quality

Up until now there has been no easy method to quantify the approximation error of large layer-based models. A very laborious method (which is not applicable for very large systems) is to build the conventional model and compare typical simulation results for macroscopic variables. Another method which also needs to simulate the corresponding conventional model is to check all errors *g*_*i*_. If all *g*_*i *_decline rapidly, the approximation quality is high. However, a large error for some *g*_*i *_(e.g. Equation 23) is not a direct evidence for poor approximation quality. If a state variable that is approximated by an equilibrium assumption for a diagonal reaction (as Equation 20) has only little influence on the state variables of the layer-based model, even a large error *g*_*i *_will not result in a large approximation error. This depends on the observability of this state variable, which is difficult to quantify and discussed elsewhere [36]. We recommend that more qualitative checks, described below, be performed in order to assess the approximation quality.

In the typical case, effector binding and the corresponding binding site phosphorylation are described in different layers. If the binding of the effector is relatively fast (as the binding of most effectors is) the approximation quality is usually high. If there are fast processes in the layer describing the binding site phosphorylation that connect all or most of the different phosphorylated species, the approximation quality is usually high as well. High parameter values for these fast processes result in high values of the exponents *a*_*i *_and therefore in a high approximation quality (Equation 26). The process of ligand binding is a candidate for such a fast process in the example system (Figure 4).

If the binding of an effector is slow, it should be considered to merge the layers describing this effector binding and the corresponding binding site phosphorylation to a single one. In this case, the erroneous equilibrium assumptions for the slow process are not introduced and do not lead to an approximation error (but do also not contribute to the model reduction).

If one is not sure about the approximation quality resulting from certain parameter combinations, one could build a conventional model of a subsystem comprising the processes belonging to the considered parameters and all processes that directly or indirectly interact with them via graded interactions. The approximation error can be analyzed quantitatively for the comparison of simulation results for macroscopic quantities of the conventional subsystem with those of a corresponding layer-based model.

This can be done for different constant input signals *x*_*i *_and all *x*_*j*_*b *(*i *≠ *j*) entering the model from the environment set to zero. The initial conditions of the conventional model have to be set such that the effect of the external (constant) *x*_*i *_entering the system is mirrored adequately. If the approximation quality is high, the analysis can be extended to time-variant input signals

(28) $$x_{i}(t)=\int_{0}^{t_{end}}f_{x_{i}}(t)dt$$

guaranteeing *x*_*i *_≥ *x*_*i*_*b *∀ *i*. External fluxes $f_{x_{i}}$ that reflect the changes in *x*_*i *_have to be included in the conventional model. These fluxes $f_{x_{i}}$ add or remove the corresponding species with phosphorylated and unoccupied binding sites from the conventional model. Note that this species is defined by *x*_*i *_- *x*_*i*_*b *in the layer-based model. The velocity with which a certain *x*_*i *_may change depends on the kinetic parameters for phosphorylation and dephosphorylation of the corresponding binding site.

#### Reduction of the model size

A typical situation in cellular signaling is that there are multiple sites within the same protein. In this case, the size of the conventional model is a product of the number of states of each site, where each possible state of a binding partner must be considered as a separate state of the site. In the layer-based model, however, each binding site that is phosphorylated has only two states (unphosphorylated and phosphorylated). The states of the binding partner are modeled in a separate module (layer) and do not increase the number of states of the original protein. Another typical case is that binding partners are phosphorylated on binding sites and bind other effectors. In this case, the argumentation above can be applied to each additional effector in such an association chain.

The exponential growth of the number of state variables with the number of binding sites and binding partners in conventional models is partly replaced by linear growth with the number of layers. Within the layers there is still exponential growth with respect to the number of molecular sites included in the layer. Both scenarios mentioned above lead to implicitly assumed equilibrium conditions for diagonal reactions in layer-based models. Each such equilibrium condition reduces the number of ODEs by one. In the small example system, one diagonal reaction (Equation 21) is assumed to be in equilibrium (Equation 20), which reduces the number of ODEs by one compared to a conventional model.

#### Stochastic simulation of layer-based models

Layer-based modeling has been introduced as a technique for deterministic modeling [3]. However, stochastic simulation of layer-based models is in principle possible.

A general problem in using stochastic simulation tools for layer-based models is that there are species that are defined by algebraic assignments. Additionally, it is crucial that the reaction parameters are allowed to be functions of species which becomes apparent when considering the correction terms. Therefore, only simulators can be used that support algebraic assignments and flexible parameter formats. Note that correcting the reaction parameters by the molecular weight of complexes (as is proposed for Moleculizer [24]) is not applicable for layer-based models as this information is not contained in layer-based models, due to the modularization.

## Implementation

ALC is a computer program that converts model definitions for layer-based models into model files in different formats as well as into documentation files. Its intuitive syntax is simple but powerful and supports the concepts of rules, macrostates and modularity.

ALC can be used offline or via a form on the ALC website [37]. ALC performs detailed consistency checks on the model definition. These checks result in informative error messages and warnings ensuring that most errors are easy to find and easy to correct. The output files of ALC are ready-to-run simulation files in the formats C MEX, MATLAB (The MathWorks), *Mathematica *(Wolfram Research) and SBML [38]. ALC also provides the model in LATEX [39,40] and plain text format to simplify its publication or presentation.

### Architecture

ALC is written in the programming language Perl [41,42]. When used offline, the model definition is read in by the script file ALC.pl which also starts the modeling procedure. The source code necessary for the generation of output files is organized in three separate Perl modules. The Perl module Procedures.pm contains procedures that guide the application through the general steps of the model generation process. The Perl module Functions.pm contains functions that are called by the procedures from the module Procedures.pm and functions that are called by other functions from the same module. The Perl module Output.pm contains procedures that generate all output files after the model generation process is finished. The form on the ALC website [37] is linked to a Perl CGI script that is running on an Apache web server [43]. This CGI script extracts the model definition from the uploaded model definition file or from the text field in which the model definition was pasted and starts the model generation. It also displays the results and provides the download links for the output files. The model generation and the generation of the output files is performed by the same Perl modules that are used offline.

**Figure 6:**
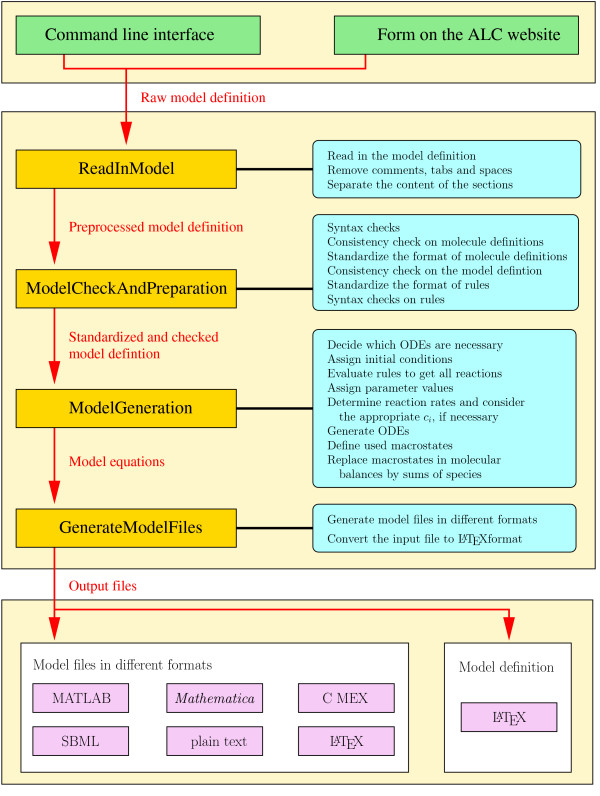
**Flow diagram of ALC**. The green boxes represent the different possibilities when starting ALC. The gold boxes represent procedures of ALC (stored in the Perl module Procedures.pm) that initiate the general processes from reading in the model definition to generating the finished model files. The red arrows represent the input and the output of the procedures. A short description of the processes that are initiated by the procedures is given in the cyan boxes. The white boxes define the principle class of the output files, while the magenta boxes define the format of the output files.

The most important steps in the generation of the output files from the model definition and the assignment of these steps to procedures of the Perl module Procedures.pm are shown in more detail as a flow diagram in Figure 6.

### Constructing the model definition

The model definition is constructed in plain text and consists of distinct sections that are encapsulated by #name and #end name, where 'name' is the name of the section. All sections may occur as often as desired in the model definition. Therefore, each layer can be defined separately, supporting the modular structure of layer-based models (see e.g. Equations 8 and 10).

**Figure 7:**
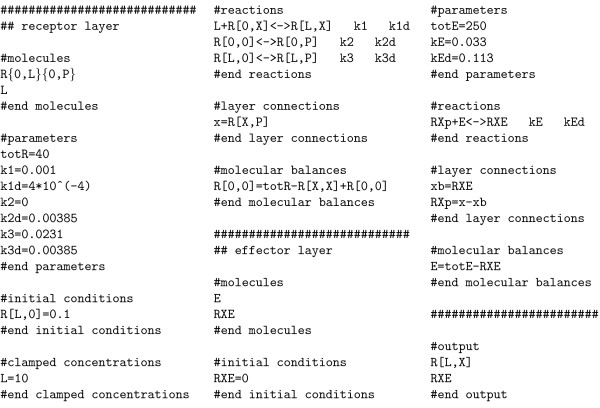
**Model definition for the example system**. Default values are assigned for all initial conditions and parameters that are not given. These default values can be freely chosen. Note that *x *≥ *xb *and *x *> 0 hold for the initial conditions of the example system when using the standard default values (see ALC user guide). When running the C MEX, MATLAB and *Mathematica *models, the simulation results for *R*[*L*, *X*] and *RXE *are visualized as they are defined as outputs. *RXp*, *x *and *xb *are also visualized as this is automatically done for all variables that are defined in the section #layer connections.

The layer-based model definition for the example system (Figure 4) is shown in Figure 7. Model definitions for other example systems, including the model definition for the layer-based model of insulin signaling with 214 ODEs [3] and its exact reduction to 51 ODEs (which is possible if some binding sites are equivalent) can be found in Additional file 3.

The syntax of ALC and the distinct sections are briefly introduced below. Detailed descriptions can be found in the ALC user guide (Additional file 1). A tutorial demonstrates the generation of the model definition for an example system in detail (Additional file 4).

#### The section '#molecules'

All molecule definitions are given in the section #molecules (Figure 7). A molecule definition consists of the molecule name that is optionally followed by the successive definition of all sites. The molecule name has to start with a capital letter which is optionally followed by an arbitrary sequence of alphanumeric symbols (letters, digits and underscores are allowed). Each site definition consists of a comma-separated sequence of all possible modifications which is encapsulated by curly brackets. A site modification is defined by a sequence of alphanumeric symbols. The definition of a site modification 'X' is forbidden, since 'X' is reserved for indicating macrostates and patterns. A site modification 'P' has a predefined meaning. It marks the site as a binding site to which effector binding is described in another layer. In this case, 'P' indicates the phosphorylated state.

As an example, a molecule definition R{0, L}{0, P} defines the molecule *R *that can have the modifications '0' and 'L' at the first site and the modifications '0' and 'P' at the second site. If a molecule is defined, its instances (e.g. species like *R*[*L*, 0] or macrostates like *R*[*X*, *P*]) can be used in other sections of the model definition.

#### Complexes

ALC only supports the simple notation of complexes introduced above. Complexes of species can be treated as species of one of the corresponding molecule definitions, or as species of new molecules that are defined for complexes of the molecules. As an example, the species *R*[*L*, 0] is the complex of the species *L *and *R*[0, 0] as this is already contained in the molecule definition R{0, L}{0, P}, whereas the dimer of R{0, L}{0, P} has to be explicitly defined, e.g. as R2{0, L}{0, P}{0, L}{0, P}.

#### The section '#reactions'

All rules and reactions are defined in the section #reactions (Figure 7). The standard format of a rule or reaction is: chemical reaction equation, tab, parameter of the forward reaction, tab, parameter of the backward reaction.

(29) $$\begin{array}{c} \begin{array}{cc} \;\overset{tab}{\overset{︷}{}} & \;\overset{tab}{\overset{︷}{}} \end{array}\\ \begin{array}{ccc} \underset{reaction\;equation}{\underset{︸}{\text{aA}+\text{bB}<->\text{cC}+\text{dD}}} & \underset{forward}{\underset{︸}{\text{parameter}}} & \underset{backward}{\underset{︸}{\text{parameter}}} \end{array} \end{array}$$

The reaction symbol is '<->' for reversible reactions and '->' or '<-' for irreversible reactions. For irreversible reactions only one parameter has to be given. ALC builds the corresponding rates following the generalized law of mass action where the parameters can be general nonlinear functions.

If a reaction describes the transition of a site modification 'P' to anything but 'P' (i.e. the dephosphorylation of a binding site to which effector binding is described in another layer), the reaction rate has to be modified using a correction term *c*_*i*_.

To guarantee that only the unoccupied fraction of binding sites with a site modification 'P' becomes dephosphorylated, ALC automatically searches the corresponding *x*_*i *_and *x*_*i*_*b *in the model definition, builds the appropriate *c*_*i *_= (*x*_*i *_- *x*_*i*_*b*)/*x*_*i *_and considers *c*_*i *_in the rate laws for dephosphorylation. As the correction terms *c*_*i *_in the reaction rates are automatically assigned by ALC, one need not include them in the reaction parameters. The algorithm for this assignment is described below.

#### The section '#layer connections'

The signal flow between the layers is defined in the section #layer connections (Figure 7). Notations of the sums of species with a phosphorylated binding site *x*_*i *_have to start with a lowercase 'x' and are optionally followed by a sequence of letters and digits. Notations of the sums of species with occupied binding sites *x*_*i*_*b *start with the notation of the corresponding *x*_*i *_and end with a lowercase 'b'. Note that notations of *x*_*i *_are not allowed to end with a lowercase 'b'.

The phosphorylation of binding sites (site modification 'P') and the corresponding effector bindings are described in different layers. In this case, the binding partners for the effectors are defined by algebraic equations, typically as differences of *x*_*i *_and *x*_*i*_*b*. This is also done in the section #layer connections. The notation of these binding partners is analogous to the notation of species, however, the binding partners do not have to be defined in the section #molecules.

#### The sections '#parameters' and '#initial conditions'

The sections #parameters and #initial conditions are used to define parameters and initial conditions, respectively (Figure 7). In contrast to species notation, parameter notations have to start with a lowercase letter (which may not be 'r' or 'x', as these letters are reserved for the notation of reaction rates and layer connections, respectively). Parameters can also be functions of other parameters (e.g. k1 = k2/k3).

Each species that is not defined by an algebraic equation requires an initial condition. An initial condition for a species is defined by an equation that assigns a value to the species. Note that initial conditions can be functions of parameters (e.g. R[0, P] = a/b+1).

Initial conditions and parameters for which no value is assigned, are set to the corresponding default values. The default values can be arbitrarily chosen (see below) and a warning is given for each omitted parameter definition or initial condition.

Setting the default value for initial conditions to zero may result in division by zero in the correction terms *c*_*i *_if no initial condition for phosphorylated species is set manually to a non-zero value. One could use a default value very close to zero (e.g. 10^-20^) to avoid this problem. During the assignment of initial conditions, one has to assure that *x*_*i *_≥ *x*_*i*_*b *and *x*_*i *_> 0 hold for all *i*. It is not possible that more binding sites are occupied than are phosphorylated, as in these cases the phosphorylation is a precondition for effector binding. Therefore, it is obvious that the condition *x*_*i *_≥ *x*_*i*_*b *∀ *i *has to be fulfilled for the initial conditions.

#### Optional sections

Conserved moieties are chemical entities that participate in a reaction system without loss of integrity and always remain in the system [6]. As an example, the sum of all species of a protein that is not degraded or synthesized is a conserved moiety. Conservation relations (molecular balances) allow for the replacement of the ODE of one species for each conserved moiety by an algebraic equation, and can be defined in the section #molecular balances. Concentrations of species can be set to a constant value (which can be a function of parameters) in the section #clamped concentrations. The section #algebraic relations allows for the assignment of arbitrary algebraic expressions (that may contain species, macrostates, numerical values and parameters) to variables whose notation is analogous to that for species. The section #remove allows for the complete removal of defined species from the reaction network and from all macrostates. This simplifies the use of rules for association reactions and is discussed in the user guide. The section #output allows for the declaration of species, macrostates, variables from the section #algebraic relations, or arbitrary algebraic expressions as outputs. All outputs are visualized in the resulting *Mathematica*, C MEX and MATLAB models and defined as *Output*_*i *_in the SBML model.

### Assignment of correction terms

In the following section, we describe the conditions that are checked in order to decide whether a correction term *c*_*i *_is necessary and if so, the algorithm to find the appropriate correction term. ALC detects the need for a correction term in the rate law of a reaction if the following two conditions are simultaneously fulfilled:

• A molecule *M *occurs on both sides of the reaction equation.

• The site modification of the molecule *M *is '*P*' on the *j*th site on one side of the reaction equation and not 'P' on the other side of the reaction equation.

As an example, these conditions are fulfilled for the second and for the third reaction in Figure 7, whereas they are not fulfilled simultaneously in the remaining reactions.

ALC uses the following algorithm to find the appropriate correction term:

1. Replace macrostates by the corresponding sums in each definition of *x*_*i*_.

2. Count for all *x*_*i *_how often the molecule *M *occurs with a site modification 'P' on the *j*th site (factors before the species are considered).

3. Take the *x*_*i *_with the highest score and use *c*_*i *_= (*x*_*i *_- *x*_*i*_*b*)/*x*_*i *_as the correction term.

The choice of the correction term is described in the ALC user guide for an example system. This algorithm is implemented much faster than described above. The first step is only performed once. The second step is performed for all molecules and positions with the site modification 'P' that occur in at least one *x*_*i *_simultaneously. This is done by building a hash (an associative array, a major data type in Perl) [41] that has the keys: molecule name and position. The values are the corresponding *x*_*i *_that have the highest score. Using this hash, the third step is trivial.

### Running ALC

There are two possible ways of running ALC. The first is to use the form on the ALC website [37], where ALC can be accessed using a browser without any additional software.

The second possibility is to download the latest release of ALC from the same website [37], from SourceForge.net [44], or to use the first release that is given in Additional file 5. Perl (freely available, e.g. [42]) is required for the offline use. For the installation of ALC it is only necessary to unpack the file 'download_ALC.zip' (or Additional file 5) in the directory where the model definition files will be stored. If ALC is installed, store the model definition in the file 'layer.alc', open a command line interface, go to the ALC directory and type

ALC.pl

followed by the return key. Another, equivalent possibility is to use

perl ALC.pl

to perform the generation of the output files. These commands execute ALC with default values, e.g. for undefined parameters or initial conditions and for the filenames of the model definition file and the resulting model files. Without changing the default values, the model definition has to be stored in the file 'layer.alc' and the notation of the resulting output files starts with 'model'. All default values can be changed in the file Config_ALC.txt or via the command line for each call of ALC separately. A detailed description of default values and command line parameters is given in the ALC user guide. Note that all options have their equivalent as text fields or select elements in the form on the ALC website [37].

### Step by step procedure: using ALC

This step by step procedure is a modification of the step by step procedure for layer-based modeling that can be applied when using ALC.

1. Identify all processes and their interactions.

2. Draw the interaction graph.

3. Deduce from the interaction graph how many layers are in the model and which processes form part of which layer. All processes that are coupled by graded interactions are part of the same layer. Processes of different layers are coupled by all-or-none interactions or do not interact.

4. Model each layer separately as if the processes of all other layers do not exist.

(a) Define all molecules in the section #molecules (e.g. R{0, L}{0, P}). The site modification (uppercase) 'P' is reserved for indicating the phosphorylation of binding sites to which the effectors bind in other layers. A different notation (e.g. lowercase 'p') has to be used to indicate the phosphorylation of other sites.

(b) Define rules and reactions, as in conventional modeling, in the section #reactions (e.g. R[0, X]+L<->R[L, X] k1 k2). Define variables in the section #algebraic relations that represent frequently used expressions in the parameter part of the reactions.

(c) Define all sums of species with phosphorylated binding sites *x*_*i *_(e.g. x = R[X, P]) and all sums of species with occupied binding sites *x*_*i*_*b *(e.g. xb = RXE) in the section #layer connections. Note that *x*_*i*_*b *has the same notation as the corresponding *x*_*i*_, followed by (lowercase) 'b'.

(d) Define the binding partners for effectors whose binding sites are phosphorylated in other layers in the section #layer connections (e.g. RXp = x-xb).

(e) Assign values to parameters in the section #parameters (e.g. k1 = 3). Undefined parameters are set to the default value (the value of the option Param) which can be defined in the file Config_ALC.txt or via the command line.

(f) Assign initial conditions in the section #initial conditions (e.g. R[0,0] = 2). Undefined initial conditions are set to the default value (the value of the option InCond) which can be defined in the file Config_ALC.txt or via the command line. The initial conditions have to guarantee *x*_*i *_> 0 and *x*_*i *_≥ *x_i_b *∀ *i*.

(g) Concentrations of species can be set to a constant value in the section #clamped concentrations (e.g. L = 10).

(h) Conservation relations (e.g. R[0,0] = totR-R[X, X]+R[0,0]) can be defined in the section #molecular balances. Using macrostates and the species whose ODE is to be replaced highly simplifies this step.

5. Define outputs (observables) to be visualized in the C MEX, MATLAB and *Mathematica *models in the section #output. All variables defined in the section #layer connections are visualized in the C MEX, MATLAB and *Mathematica *models without declaring them as outputs. This can be disabled by changing the default value (the value of the option OutLC) which can be defined in the file Config_ALC.txt or via the command line.

6. Store the model definition in a file, e.g. 'layer.alc', which is the default file name for the model definition file.

7. Run ALC offline or use the form on the ALC website.

### Output files

The resulting model is given in MATLAB (default: modelM call.m and modelM.m), C MEX (default: modelC call.m, modelC mdl.mdl and modelC.c), *Mathematica *notebook (default: model.nb) and *Mathematica *input file (default: model.mma.m) formats as directly executable files. They include the visualization of user-defined output variables and – in the default case – all variables defined in the section #layer connections.

The core of the C MEX model is a MATLAB S-function written in C, which results in a much faster simulation of the model compared to the standard MATLAB format. A recent release of MATLAB (earliest version successfully tested: 7.0.4.352) is required for using the C MEX models. The *Mathematica *input file contains the same code as the notebook file but simplifies the integration of layer-based models into existing mathematica code.

The model is also given in SBML (default: model.xml), LATEX (default: model.tex) and plain text (default: model.txt) formats, which allows for the direct usage of model equations for presentations or publications. Additionally, the model definition is converted to the LATEX format (default: model.input.tex).

Note that the SBML standard [38] allows for explicit algebraic assignments, which are used in layer-based models. However, some SBML-supporting tools will not work with layer-based SBML models as these tools do not support algebraic assignments. An important tool that works with layer-based SBML models is MathSBML [45] (release 2.7.0.3 or higher).

## Results and discussion

### ALC: Functionality and performance

#### Simplification of the model generation

ALC converts model definitions given in a simple but powerful rule-based syntax to computational models in different formats, as well as documentation files. The main benefit of ALC is that it dramatically simplifies layer-based modeling and reduces the risk of creating erroneous model equations.

The assignment of the correction terms *c*_*i *_to dephosphorylation rates is one of the more difficult and error-prone steps in manual layer-based modeling. ALC performs these assignments automatically, such that errors in this step are avoided. This strengthens the analogy of layer-based modeling to conventional modeling and rule-based modeling as the reaction network within layers can now be defined using rules without considering correction terms.

ALC also supports the usage of macrostates. This highly simplifies the definition of the signals between the layers (*x*_*i *_and *x*_*i*_*b*) as well as the definition of conservation relations and the use of enzyme kinetics for rules and reactions.

#### Portability of models

In many cases, it is comfortable to have the model in different formats. As an example, SBML [38] is becoming the *de facto *standard for model representation in systems biology. Though by far not all modeling and simulation projects use SBML, it is often desired to provide SBML models for publications. In many cases, the model equations in plain text are part of the manuscript or are provided as supplementary material. ALC automatically exports the model to several formats including SBML and provides the model equations in LATEX format as well as in plain text format.

The manual format conversion of models is probably the major reason for errors in published model descriptions. Automation of this step, as provided by ALC, is not only convenient but also lowers the probability of errors in the provided models.

#### Application range of ALC

ALC is optimized for building layer-based models. It supports features of layer-based modeling that are not present in conventional or rule-based modeling, and that are not supported by other tools. ALC is also well suited for conventional modeling of reaction networks. Although the functionality of ALC is related to other tools that also allow for rule-based modeling [20-26], the application range is different. Note that rule-based conventional models can be built using ALC. However, due to limitations in the description of complexes, ALC is not as well suited for this task as e.g. BioNetGen [20-22]. ALC shows its optimal performance when building reduced modular models according to the layer-based approach.

#### Computational aspects

The output files for small layer-based models are generated in less than one second on a desktop PC. The generation of larger layer-based models that can correspond to extremely large conventional models is also very rapid. As an example, the output files for the layer-based model of insulin signaling (214 ODEs) that replaces a conventional model with 1.5·10^8^ ODEs [3] are generated in about one second on a desktop PC. A few thousand ODEs are usually generated within seconds.

The model size in the offline version of ALC is only restricted by CPU time, disk space and memory capacity. The online version of ALC which is accessible via a form on the ALC website [37] provides full functionality, but is restricted to model definitions that define no more than 500 species. This is done to keep the traffic and the processor load on the server at a reasonable size.

### The choice of the modeling method

An important question one has to answer at the beginning of the modeling process is that of which modeling method is the most suited for the considered system. A principle decision is necessary between stochastic and deterministic simulation.

If stochastic simulation is chosen, it depends on the model size, as well as on the branching of the network and on the number of simulation runs as to whether on-the-fly or generate-first modeling is more suited. Note that layer-based modeling is also suited for stochastic simulation. In the following paragraph, we give a rule of thumb for choosing the appropriate deterministic modeling technique.

The exact techniques should be used if it is possible and applicable. Whether one should use conventional modeling, rule-based modeling or exact model reduction simply depends on the size of the resulting model and on the number of necessary simulations. Additional assumptions (e.g. assuming a temporal order of processes) that are only made to keep the numbers of species and reactions low but have no physiological justification should be avoided. Such assumptions may lead to a nearly unpredictable approximation error [5,18].

If the conventional model (which may be the result of rule-base modeling) is too large for efficient simulation or parameter estimation, and if the exactly reduced model is also too large or is difficult to generate, layer-based modeling is a powerful possibility. In addition, the layer-based formalism is especially suited for comparing many model variants, as in many cases only a few equations in a single layer have to be changed. Note that rule-based modeling is also possible for layer-based models when using ALC. If the layer-based model is still too large for efficient simulation or parameter estimation, the layer-based model should be subjected to exact model reduction. In this case, the exact model reduction is performed on each layer separately. This combination of layer-based modeling and exact model reduction results in the fewest ODEs without the introduction of an additional approximation error as introduced by layer-based modeling.

## Conclusion

The layer-based approach allows for a highly reduced macroscopic description of signaling systems with inherent combinatorial complexity. It is an approximative, but accurate modeling technique that results in a reduced model with a pronounced modular structure. An interaction graph which represents the interactions between the processes defines the modularity of the model. The resulting modules, called layers, are connected in a standardized way and can be modeled separately from each other once their connections are defined.

ALC highly simplifies the generation of layer-based models. The tool can be used offline or via a form on the ALC website. The simple but powerful syntax of the model definition supports the concepts of modularity, rules and macrostates. The model definition is divided into distinct sections and can be structured to mirror the modularity of the model. ALC provides the resulting dynamic models in different formats (C MEX, MATLAB, *Mathematica *and SBML) as ready-to-run simulation files, and provides documentation files that simplify the presentation and publication of the models.

## Availability and requirements

Project name: ALC

Project home page: 

ALC on SourceForge.net: 

Operating system(s): Platform independent

Programming language: Perl

License: GNU Lesser General Public License

Any restrictions for use by non-academics: None

## Authors' contributions

MK wrote the Perl scripts and drafted the manuscript, EDG initiated and supervised the study.

## Supplementary Material

Additional file 1 **ALC user guide.** The ALC user guide describes the syntax and the usage of ALC in detail. There is also a section describing frequently occurring problems and their solutions. Click here for file

Additional file 2 **Model definitions to illustrate the advanced strategies.** Several layer-based model definitions illustrating the concepts discussed in the "advanced strategies" section are given in this file. Click here for file

Additional file 3 **Example models.** Several layer-based model definitions for example systems are given in this file. Click here for file

Additional file 4 **ALC tutorial.** The ALC tutorial extensively describes the modeling of an example system. Click here for file

Additional file 5 **ALC for offline use.** The first release of ALC is given in this file. Click here for file
