# Supplementary material for: ALC: automated reduction of rule-based models
Source: BMC Syst Biol. 2008 Oct 31;2:91. doi: 10.1186/1752-0509-2-91 (PMC2636783; doi:10.1186/1752-0509-2-91)
Supplement: Additional file 1 — ALC user guide. The ALC user guide describes the syntax and the usage of ALC in detail. There is also a section describing frequently occurring problems and their solutions. [file 1752-0509-2-91-S1.pdf]

Automated Layer Construction (ALC) - User Guide

Markus Koschorreck and Ernst Dieter Gilles

Release 1.0, October 2008

Contents

1 About **2**

2 The model definition **2**
2.1 Molecules and species . . . 2
2.2 Reactions and rules . . . 3
2.2.1 Reactions . . . 3
2.2.2 Rules . . . 3
2.2.3 Symmetric reaction rules . . . 4
2.2.4 Constraints on rule definitions . . . 4
2.2.5 Complex association rules . . . 5
2.2.6 Enzyme kinetics for rules . . . 6
2.3 Layer connections . . . 6
2.4 Correction terms . . . 6
2.5 Parameters . . . 7
2.6 Initial conditions . . . 7
2.7 Clamped concentrations . . . 7
2.8 Conservation relations . . . 8
2.9 Algebraic assignments . . . 8
2.10 Model output . . . 8

3 Order of assignments **8**
3.1 Order of sections . . . 8
3.2 Order within sections . . . 9

4 Syntax restrictions **9**
4.1 Tabs and spaces . . . 9
4.2 Beginning of lines and comments . . . 9
4.3 Species and other variables . . . 9
4.4 Complexes . . . 9
4.5 Layer connections . . . 10
4.6 Parameters . . . 10
4.7 Numerical values . . . 11
4.8 Stoichiometric coefficients and multiplication . . . 11
4.9 Compartments . . . 11

5 Consistency checks **11**
5.1 Molecule definitions . . . 12
5.2 Used variables . . . 12
5.3 Initial conditions and parameters . . . 12
5.4 Rules and reactions . . . 12
5.4.1 Minimal syntax restrictions . . . 12
5.4.2 Strict syntax restrictions . . . 12
5.5 Layer connections . . . 12

6 Installation **12**

7 Running ALC **13**

8 Model files **13**

9 Frequent problems 14
9.1 Division by zero 14
9.2 Negative concentrations 14
9.3 Command line 14
9.4 MATLAB and C MEX 14
9.5 C MEX 14
9.6 *Mathematica* 14
9.7 SBML 14

List of Tables

1 Syntax restrictions for the notation of entities 15
2 Regular expressions for entities 15
3 Command line parameters and default values 16

1 About

ALC is a computer program that performs automated layer-based modeling following a model definition with a simple but powerful syntax. Conventional mechanistic models can also be easily built. However, its focus is on simplifying and automating the construction of models that are according to the layer-based formalism [1]. ALC supports the concepts of modules, rules and macrostates, which simplifies the modeling procedure dramatically. The finished model is given as ready-to-run simulation files in the formats C MEX, MATLAB, *Mathematica* and SBML. Output variables can be freely defined, their visualization is already included in the C MEX, MATLAB and *Mathematica* model files.

A thorough consistency check is performed on the model definition. In the case of errors in the model definition detailed warnings and error messages are given that indicate the nature and location of the errors. ALC can be used offline or via a form on the ALC website. For the offline use the freely available programming language Perl (www.cpan.org) is required.

In this user guide, we show the syntax and the usage of ALC in detail. We describe the consistency checks that are performed on the model definition and frequently occurring problems as well as their solutions.

2 The model definition

The model definition is given as plain text in a model definition file (default: 'layer.alc') or can be pasted in the form on the ALC website. It is structured into distinct sections and mirrors the modularity of the layer-based model.

The distinct sections are enclosed by the commands `#name` and `#end name`, where 'name' is the name of the section. Everything that is not included within the lines `#name` and `#end name` is ignored by ALC. Each section can be split into an arbitrary number of blocks that are encapsulated by `#name` and `#end name`. Therefore, different layers can be defined independently from each other. In the following, we refer to the sum of all blocks of a section if we refer to this section.

The section `#molecules` contains all molecule definitions. The section `#reactions` contains all rules and reactions of the system. The section `#layer connections` defines signal flows and binding partners that constitute the connectivity between the different layers. The section `#algebraic relations` can contain arbitrary explicit algebraic assignments, e.g. for variables defining enzyme activity or for variables used as outputs. The section `#output` defines which species, macrostates or other variables shall be visualized by the model files. Arbitrary algebraic expressions may also be declared as outputs.

The section `#molecular balances` can be used to define conservation relations. This reduces the systems order if there are conserved moieties. The sections `#parameters` and `#initial conditions` are used to define parameters and initial conditions, respectively. Each section (except for `#molecules` and `#reactions`) can be omitted if it is not used.

Comments can be added anywhere in the model definition. The symbols '#' and '%' label the rest of the line as a comment. Note that lines intended to be a comment but starting with '#name' are interpreted as the start of the section `#name` if this section exists in ALC. Using '%' as the comment symbol avoids this problem. Comments can be whole lines or can be located after some definition.

2.1 Molecules and species

All molecules have to be defined in the section `#molecules`. A molecule definition consists of the molecule name followed by the successive definition of all sites. Definitions of molecules have to match the syntax restrictions shown in Table 1. The molecule

name starts with an uppercase letter that is optionally followed by an arbitrary sequence of alphanumeric symbols (allowed are: letter, digit and the underscore character). A site definition consists of the comma-separated sequence of all possible modifications enclosed by curly brackets. ALC orders all possible modifications of each site definition according to the ASCII table while the order of the sites remains unchanged.

The notation of a modification consists of a sequence of alphanumeric symbols. The notation 'X' for a modification is forbidden, as this is reserved for patterns in rules and for macrostates. The modification 'P' marks the site as a binding site to which effector binding is described in another layer. As defined by the layer-based formalism, 'P' indicates just phosphorylation, without distinguishing whether an effector is bound or not [1].

As an example, imagine a receptor R with three sites. The first is a ligand binding site which can be occupied (in the state 'L') or unoccupied (in the state '0'). The second site is a binding site to which effector binding is described in another layer and that can be unphosphorylated (in the state '0') or phosphorylated (in the state 'P'). The third site is a regulatory phosphorylation site and can be unphosphorylated (in the state '0') or phosphorylated (in the state 'p'). The molecule definition of the receptor R is given as `R{0,L}{0,P}{0,p}`.

The notations of species and macrostates start with the molecule name which is followed by the comma-separated list of site modifications that is encapsulated by squared brackets. As an example, the species of R without bound ligand and with both sites being phosphorylated is denoted as $R[0, P, p]$.

Molecule definitions of molecules that only exist in a single modification just consist of the molecule name, e.g. `A`. The single species of A is denoted as A. Alternatively, the molecule name may be followed by the definition of the sole site enclosed by curly brackets, e.g. `A{1}`. In this case the single species is denoted as $A[1]$.

2.2 Reactions and rules

Rules and reactions are defined in the section `#reactions`.

2.2.1 Reactions

A typical reaction is given as

$$\underbrace{\texttt{aA+bB...<->cC+dD...}}_{\textit{reaction equation}}\overbrace{\quad}^{\textit{tab}}\underbrace{\texttt{k1}\overbrace{\quad}^{\textit{tab}}\texttt{k1d}}_{\textit{parameters}}$$

where A, B, C and D are molecular species, a, b, c and d are stoichiometric coefficients and $k1$ and $k1d$ are the parameters of the forward and backward reactions, respectively. According to a generalized law of mass action, the reaction rate is given as

$$r = k1 \cdot A^a \cdot B^b ... - k1d \cdot C^c \cdot D^d ...,$$

where A, B, C and D are the concentrations of the corresponding species.

Note that one side of the reaction equation may be empty. This allows for the realization of synthesis and degradation reactions. In this case, the part of the rate equation corresponding to the empty side consists just of the reaction parameter.

All reactions have to match the general format of a reaction. This is: left hand side of the chemical reaction equation, reaction symbol ('<->','->' or '<-'), right hand side of the chemical reaction equation, one or more tabs, parameter of the forward reaction (from the left hand side to the right hand side), one or more tabs, parameter of the backward reaction (from the right hand side to the left hand side). Note that for irreversible reactions (reaction symbol '->' or '<-') only one parameter has to be given. The declaration of two however, is also allowed. In this case, the parameter of the reaction that does not take place is ignored.

The parameters $k1$ and $k1d$ can be replaced by arbitrary algebraic expressions using basic arithmetics, potentiation and brackets. Numerical values, species, variables that are defined in the sections `#layer connections` (chapter 2.3) and `#algebraic relations` (chapter 2.9), macrostates and parameters can be used in the parameter part of reactions. Macrostates in the parameter part are interpreted as the sums they represent.

ALC generates the rates r_i and all necessary ODEs from the given reactions. Note that brackets around the parameter expressions are generated automatically if this is necessary. During the generation of the reaction rates, ALC automatically adds the right correction term $c_i = (x_i - x_i b)/x_i$ to each reaction rate for the dephosphorylation of sites with a site modification 'P' (see chapter 2.4). This ensures that only phosphorylated binding sites without bound effector are dephosphorylated.

2.2.2 Rules

A rule represents a class of reactions with common properties [2–4]. In particular, all reactions of this class are parameterized by the same kinetic parameters. The hallmark of rules is that they must contain at least one macrostate in the reaction equation. In this case, the macrostates are interpreted as patterns and represent each of the corresponding species individually.

Note that the sole occurrence of macrostates in the parameter part of a reaction does not result in a rule, as at this position macrostates represent the sums of all corresponding species.

As an example, for `R{0,L}{0,P}{0,p}` the rule

```
R[0,X,X]+L<->R[L,X,X]     k0    k1
```

represents the reactions

```
R[0,0,0]+L<->R[L,0,0]     k0    k1
R[0,0,p]+L<->R[L,0,p]     k0    k1
R[0,P,0]+L<->R[L,P,0]     k0    k1
R[0,P,p]+L<->R[L,P,p]     k0    k1.
```

Rules may also be used for association reactions, as exemplified for dimerization in the rule

```
Rmon[X]+Rmon[X]<->Rdim[X,X]     k0     k1
```

which for Rmon{0,L} and Rdim{0,L}{0,L} is evaluated to

```
Rmon[0]+Rmon[0]<-> Rdim[0,0]  k0  k1
Rmon[0]+Rmon[L]<-> Rdim[0,L]  k0  k1
Rmon[L]+Rmon[0]<-> Rdim[L,0]  k0  k1
Rmon[L]+Rmon[L]<-> Rdim[L,L]  k0  k1.
```

Stoichiometric coefficients can be used in rules. As an example, the rule

```
2 Rmon[X]<->Rdim[X,X]     k0    k1
```

is evaluated leading to the same results as above.
Note that compared to the binding of L, binding sites for the aggregation of two larger molecules (e.g. the dimerization of $Rmon$) do not have to be defined. In this case, the complex (e.g. $Rdim$) is defined as a new molecule with a site definition that is the sequence of the site definitions of the reactants.

2.2.3 Symmetric reaction rules

ALC deals with the problem of symmetric reaction rules correctly. In the example above, the second and the third reaction are symmetric to each other. If the two sites on $Rdim$ are really equivalent, $Rdim[0, L]$ and $Rdim[L, 0]$ are indistinguishable species. $Rdim$ with one bound L is represented by $Rdim[0, L]+Rdim[L, 0]$. Therefore, the rate of association of $Rmon[0]$ and $Rmon[L]$ is twice the nominal rate constant $k0$.
This choice of parameterization differs from the one used by BioNetGen 2 [5], where homodimerization is parameterized with 0.5 times the nominal rate constant. Both solutions are equivalent but differ by a constant factor of two in the parameterization.

2.2.4 Constraints on rule definitions

Two options can be chosen for constraints on rule definitions (command line option `StrictRS` or choice in the form on the ALC website).
Minimal syntax restrictions: If only minimal syntax restrictions are intended (`StrictRS=0`), the only restriction is that (from the left to the right on both sides of the reaction equation) corresponding sites with a modification 'X' need to have the same site definition. Overhanging sites with a modification 'X' are allowed. As an example, the rule

```
Rdim[X,0]+R2[X,X]<->R4[X,X,0,X]     k0     k1
```

with Rdim{0,L}{0,L}, R2{0,L}{0,P} and R4{0,L}{0,L}{0,L}{0,P} is correct when choosing `StrictRS=0`. The first and second sites with modifications 'X' both have the site definition {0,L}, the third sites with modifications 'X' both have the site definition {0,P}.
Only imposing this single restriction allows for a great flexibility of rule definitions. However, unintended errors may occur. If different sites of the same molecule have the same site definition, it can easily happen that the positions of two sites are exchanged in the rule definition. It could be possible that in the rule above the modification of the second and third sites of $R4$ are exchanged while the desired rule is

```
Rdim[X,0]+R2[X,X]<->R4[X,0,X,X]     k0     k1.
```

Strict syntax restrictions: If more restrictive constraints are chosen (`StrictRS=1`), the sequences of molecule definitions from the left to the right on both sides of the reaction equation have to be identical. In addition, the positions of 'X' from the left to the right on both sides of the reaction equation have to match. This means that the modification of each position has to be 'X' on both sides or anything but 'X' on both sides. As an example,

```
Rdim[X,0]+R2[X,X]<->R4[X,0,X,X]     k0     k1
```

with Rdim{0,L}{0,L}, R2{0,L}{0,P} and R4{0,L}{0,L}{0,L}{0,P} is correct, as the sequence of molecule definitions of each side is {0,L}{0,L}{0,L}{0,P}, and the positions of 'X' do match. The rule

```
Rdim[X,0]+R2[X,X]<->R4[X,X,0,X]     k0     k1
```

is not allowed as the positions of 'X' do not match. However, this rule is allowed when choosing `StrictRS=0`.
In most cases, the number of sites will be the same on both sides of the rule. However, sequences of molecule definitions can be of different lengths if the shorter one is fully contained in the large one from the left to the right and if the positions of 'X' do match. It is also allowed that the numbers of 'X' on both sides of the reaction differ. As an example, the rules

```
Rdim[X,0]+R1[X]<->Rdim[X,L]     k0     k1
```

```
Rdim[X,0]<->    k0    k1
```

with R1{0,P} and Rdim{0,L}{0,L} are allowed. Such reactions could be used to realize degradation or synthesis reactions.

2.2.5 Complex association rules

A very strong restriction for rule definitions is that corresponding sites with a modification 'X' need to have the same site definitions. Rules sometimes desired define the association of a ligand $L1$ to a scaffold or a receptor in the same layer.

```
R[X,0]+L1[X]<->R[X,X]    k1    k2
```

This rule can be evaluated to reactions if the second site of the receptor has the same site definition as the ligand and if the option StrictRS=0 is chosen. This implies that a possible site modification of the ligand $L1$ has to be '0' and that the evaluation of the rule to reactions leads to reactions of the type

```
R[X,0]+L1[0]<->R[X,0]    k1    k2.
```

This is in most cases not the desired result as the reactions are degradation reactions for $L1[0]$.
A possible solution of this problem is to define the molecule L in a slightly different way than it may be expected and to remove species completely from the expanded system. Removing species means that all reactions of these species are removed and that theses species do not occur in macrostates. We illustrate this on a example. The model definition

```
#molecules
R{0,1}{0,L1,L12,L123}
L1{0,L1,L12,L123}
#end molecules

#reactions
R[X,0]+L1[X]<->R[X,X]   k1   k2
#end reactions
```

results in the reactions

$$
\begin{aligned}
R[0,0] + L1[L1] &\leftrightharpoons R[0,L1] \quad k1 \quad k2\\
R[0,0] + L1[L12] &\leftrightharpoons R[0,L12] \quad k1 \quad k2\\
R[0,0] + L1[L123] &\leftrightharpoons R[0,L123] \quad k1 \quad k2\\
R[1,0] + L1[L1] &\leftrightharpoons R[1,L1] \quad k1 \quad k2\\
R[1,0] + L1[L12] &\leftrightharpoons R[1,L12] \quad k1 \quad k2\\
R[1,0] + L1[L123] &\leftrightharpoons R[1,L123] \quad k1 \quad k2.
\end{aligned}
$$

if one removes the species $L1[0]$ completely from the system. In addition, the macrostate $L1[X]$ then is defined as

$$L1[X] = L1[L1] + L1[L12] + L1[L123].$$

This makes sense if one considers the species $L1[L1]$ as the molecule $L1$ having the configuration 'L1' which indicates that it is not modified (intuitively one would denote this species as $L1[0]$).
Complete removal of a species, i.e. removal of all reactions where it occurs and not considering it in all macrostates can be achieved by using the section #remove. Including

```
#remove
L1[0]
#end remove
```

into the model definition completely removes $L1[0]$ from the system and results in the reactions above.
If $L1$ can only bind to the phosphorylated binding site on R, this can be described by an analogous model definition.

```
#molecules
R{0,1}{0,p,L1,L12,L123}
L1{0,p,L1,L12,L123}
#end molecules

#reactions
R[X,p]+L1[X]<->R[X,X]   k1   k2
#end reactions

#remove
L1[0]
L1[p]
#end remove
```

In this case, the species $L1[0]$ and $L1[p]$ are only defined to allow for the evaluation of the rule and afterwards completely removed. The resulting reactions are

$$
\begin{aligned}
R[0,p] + L1[L1] &\leftrightharpoons R[0,L1] \quad k1 \quad k2\\
R[0,p] + L1[L12] &\leftrightharpoons R[0,L12] \quad k1 \quad k2\\
R[0,p] + L1[L123] &\leftrightharpoons R[0,L123] \quad k1 \quad k2\\
R[1,p] + L1[L1] &\leftrightharpoons R[1,L1] \quad k1 \quad k2\\
R[1,p] + L1[L12] &\leftrightharpoons R[1,L12] \quad k1 \quad k2\\
R[1,p] + L1[L123] &\leftrightharpoons R[1,L123] \quad k1 \quad k2
\end{aligned}
$$

and the macrostate L1[X] is defined as

$$L1[X] = L1[L1] + L1[L12] + L1[L123]$$

which is the desired result.
If macrostates are set into the section #remove, all corresponding species are removed. Obviously, this makes only sense for molecules with more than one site.

2.2.6 Enzyme kinetics for rules

Rules can also be parameterized by generalized mass actions kinetics in the same way as reactions. This is demonstrated for the degradation of a molecule M that has the molecule definition `M{0,1,...,q}` which represents the species $M[i]$, $i \epsilon [0, q]$. The degradation follows macroscopic Hill-kinetics for $M[X]$.

$$M[X] \rightarrow \qquad \frac{r_{max} \cdot M[X]^{n-1}}{M[X]^n + K_M^n}$$

This results in the rates

$$r_i = \frac{r_{max} \cdot M[i] \cdot M[X]^{n-1}}{M[X]^n + K_M^n}$$

which together are

$$r_{tot} = \sum_{i=0}^{q} r_i = \frac{r_{max} \cdot M[X]^n}{M[X]^n + K_M^n}.$$

It can be seen that the macroscopic rate r_{tot} is allocated correctly on the species $M[i]$.

$$r_i = \frac{M[i]}{M[X]} \cdot r_{tot}$$

Note that macroscopic enzyme kinetics for $M[X]$ makes more sense than assuming Hill-kinetics for each species separately, e.g. by assuming

$$r_i = \frac{r_{max} \cdot M[i]^n}{M[i]^n + K_M^n}.$$

Due to the nonlinearity of these kinetics, the sums of all r_i in both cases are not equal. In macroscopic Hill-kinetics for $M[X]$, the parameter r_{max} is the maximal total conversion rate of the enzyme that degrades M. If one assumes Hill-kinetics for each species separately, the maximal total rate depends on the number of possible modifications of M and equals $(q+1) \cdot r_{max}$. In addition, K_M is the concentration of half-maximal total conversion only in macroscopic Hill-kinetics for $M[X]$.

2.3 Layer connections

The signal flow between the layers is defined in the section `#layer connections`. The notations of the sums of species with phosphorylated binding sites x_i have to start with a lowercase 'x' that is optionally followed by an arbitrary sequence of letters and digits. The corresponding sums of species with occupied binding sites x_ib have the same notation as x_i but with an appended lowercase 'b' (see Table 1). Note that the letter 'b' is not allowed as the last character of the notation of x_i. The species that act as binding partners in binding reactions are to be defined as differences of x_i and x_ib (e.g. `RXp=x-xb`). The notations of these species have to match the restrictions on species notation (Table 1). They start with a capital letter that is optionally followed by an arbitrary sequence of alphanumeric symbols (letter, digit and the underscore). Comma-separated sequences of alphanumeric symbols encapsulated by one pair of squared brackets can be optionally appended. These variables do not have to be defined in the section `#molecules`.
Both x_i and x_ib have to be defined before defining their difference, otherwise this causes problems when using the C MEX, MATLAB or SBML models.

2.4 Correction terms

During the generation of reaction rates from reactions, the right correction term $c_i = (x_i - x_ib)/x_i$ is automatically considered in dephosphorylation rates of binding sites with a site modification 'P'. This automatic assignment is performed if species with the same molecule name are on the left and right hand sides of the reaction equation and if the one has the modification 'P' at a specific site and the other not. The right x_i is chosen by counting for all x_i, how often the 'P' that is to be removed occurs at the corresponding site of the corresponding molecule. Macrostates in the definitions of x_i are internally replaced by the sums they represent, before this is done. The x_i with the highest score is the x_i ALC uses for constructing the correction term c_i that is used in the dephosphorylation rate for this site.
Integer factors in x_i before species and macrostates (e.g. $2 * R[0, P]$) are considered. If brackets with a leading factor encapsulate an expression, this factor is not considered during the scoring (e.g. the factor 2 is ignored in $2 * (R1[X, P] + R[X, P])$, use $2 * R1[X, P] + 2 * R[X, P]$ instead).
As an example for the assignment of correction terms, the dephosphorylation of a receptor `R{0,P}{0,P}` with two binding sites is discussed.

```
#layer connections
x1=R[X,P]
x2=R[P,X]
#end layer connections

#reactions
R[0,0]<->R[0,P]   k1   k2
#end reactions
```

The molecule R is to be dephosphorylated at the second site as the modification of the second site of R is 'P' on the right hand side and '0' on the left hand side. Therefore, it is analyzed how many species of R with the modification 'P' at the second site occur in $x1$ and $x2$. $x1$ and $x2$ are expanded to $x1 = R[0, P] + R[P, P]$ and $x2 = R[P, 0] + R[P, P]$. $x1$ contains two species of R with the modification 'P' at the second site, $x2$ contains only one such species. Therefore, $x1$ is the x_i

that is considered by ALC in the dephosphorylation rate. The rate corresponding to the reaction above is given as

$$r_0 = k1 \cdot R[0,0] - k2 \cdot \frac{x1 - x1b}{x1} \cdot R[0,P].$$

Note that for the reaction

```
R[P,0]<->R[P,P]    k1    k2
```

the same correction term is chosen as the 'P' at the first site is not removed.

The automatic assignment of correction terms works highly robust in normal scenarios. If atypical operations (e.g. division, subtraction, multiplication by factors with exponents) are performed in x_i, the automatically assigned correction terms should be checked carefully. However, these operations are usually not necessary because x_i is intended to correspond to sums of species with phosphorylated binding sites.

If the conditions for the need of a correction term are fulfilled for more than one molecule or site in a single reaction, or if there is no unique x_i with the highest score, ALC gives an error message but proceeds with the modeling procedure. In this case, one of the possible c_i or a general placeholder is taken as the correction term. This correction term has to be checked and may be replaced manually in the completed model files. However, these problems usually result from errors in the model definition.

2.5 Parameters

Values for parameters can be assigned in the section `#parameters` (e.g. `k1=0.1`). Parameter notations start with a lowercase letter (which may not be 'x' or 'r') that is optionally followed by an arbitrary sequence of letters and digits. The letters 'x' and 'r' are not allowed as start of the notation, as 'x' is reserved for the notation of the sums of species with phosphorylated binding sites x_i and 'r' is reserved for the internal notation of reaction rates. For syntax restrictions on parameters see Table 1. Note that parameter assignments can also be functions of parameters (e.g. `k2=2*k1`).

Parameters can be used in each section except for the section `#molecules`. In the section `#reactions` they can only be used in the tab-separated fields after the definition of the chemical reaction equations where the reaction rates are parameterized. Therefore, parameters cannot be stoichiometric coefficients.

ALC parses the model definition to find all used parameters. Those that are not defined in the section `#parameters` are set to the default value and a warning is given. This default value is set via the command line option `Param` (see chapter 7).

Only necessary parameter assignments are processed by ALC. Unnecessary parameter definitions are ignored and a warning is given. As an example, `k=a/b` is processed if k is used somewhere outside the section `#parameters`. The parameters a and b then do not have to be used somewhere else. The chain of assignments `a=c`, `k=a/b` is processed if k is used outside the section `#parameters`. Longer chains are also allowed.

In the model files, all parameter assignments where other parameters occur are moved to the end of the parameter list, however in the order they are defined. All other parameter assignments are sorted alphanumerically and placed above.

Numerical values in decimal notation (e.g. 0.0023) or scientific notation (e.g. 2.3*10^(-3) or 2.3e-3) can be used for the definition of parameter values (see chapter 4.7).

2.6 Initial conditions

Species that are defined by a molecule definition and whose value is not defined by an algebraic assignment need an initial condition. Initial conditions for species are assigned in the section `#initial conditions` (e.g. `R[L,0]=10`). Note that initial conditions can be functions of parameters.

If an initial condition is necessary but not assigned, the default value for initial conditions is taken and a warning is given. There is also a warning if an assigned initial condition is not necessary. The default value is set via the command line option `InCond` (see chapter 7). Note that `InCond=0` is in most cases not optimal, as $\frac{x_i - x_i b}{x_i} = \frac{0}{0}$ may occur in the reaction rates for the dephosphorylation of binding sites. Therefore, the assignment of initial conditions has to guarantee $x_i > 0 \ \forall \ i$. To achieve this, assign some initial conditions for phosphorylated species manually, or use a default value very close to zero instead. To avoid negative concentrations of binding partners, the initial condition also have to guarantee $x_i \geq x_i b \ \forall \ i$.

Numerical values in decimal notation (e.g. 0.0023) or scientific notation (e.g. 2.3*10^(-3) or 2.3e-3) can be used for the definition of initial conditions (see chapter 4.7).

2.7 Clamped concentrations

If a concentration of a species has to be constant, it can be clamped (i.e. set equal to a numerical value or a function of parameters, e.g. `L=10`) in the section `#clamped concentrations`. In this case, no ODE is generated for this species. Note that clamping the concentration of a protein species in most cases (i.e. if there is no conservation relation) results in an overall concentration of this protein that is not constant. In the example system in the manuscript, clamping L leads to a non-constant sum $L + R[L, X]$.

2.8 Conservation relations

The total concentration of conserved moieties is constant, if the volume is constant. In the section `#molecular balances`, one of the corresponding species for each conserved moiety can be defined by an explicit conservation relation. No ODE is generated for these species. Considering `R{0,L}{0,P}`, an example for a conservation relation is

```
R[0,0]=totR-R[0,P]-R[L,0]-R[L,P]
```

where *totR* is a parameter that defines the total amount of *R*. Note that a conservation relation can be expressed using macrostates and the species whose concentration is defined by this conservation relation. Therefore, the two alternatives

```
R[0,0]=totR-R[X,X]+R[0,0]
R[0,0]=totR-(R[X,X]-R[0,0])
```

are also allowed. The used macrostates are replaced by the corresponding sums of species. Then, the species whose concentration is defined by this conservation relation is removed from the right hand side of the assignment, including all arithmetic operators ('+','-','*') and factors directly before the species. From this it follows that using

```
R[0,0]=totR-R[X,X]
```

leads to the same result as the assignments above.
The automatic removal of the species that is defined by the conservation relation from the right hand side of the assignment allows for the usage of macrostates. Therefore, ALC highly simplifies the definition of conservation relations.
As discussed in chapter 2.5, parameter notations may not start with 'x' or 'r'. Therefore, choosing parameter notations such as *rTot* is not allowed. Those who prefer an uppercase species instead of a parameter (in this case *totR*) may define a species (e.g. *Rtot*) in the section `#molecules` (chapter 2.1) and clamp its concentration in the section `#clamped concentrations` (chapter 2.7). Note that a numerical value or an arbitrary algebraic expression can also be used instead of a parameter.

2.9 Algebraic assignments

Arbitrary explicit assignments containing e.g. species, macrostates, numerical values and parameters can be defined in the section `#algebraic relations`. Variables defined in this section may simplify the usage of recurrent expressions in the parameterization of reaction rates or the output of lengthy algebraic expressions. The notations of variables that are defined in this section have to match the restrictions on species notation (Table 1). They start with a capital letter that is optionally followed by an arbitrary sequence of alphanumeric symbols (letters, digits and the underscore character). Comma-separated sequences of alphanumeric symbols encapsulated by one pair of squared brackets can be optionally appended. Variables that are defined in this section (just by assigning an algebraic expression to them) do not have to be defined in the section `#molecules`.
If a new algebraic relation is a function of a variable defined by another algebraic relation, the new assignment has to be placed below the other one.

2.10 Model output

Species, macrostates or arbitrary algebraic expressions can be defined as model outputs (observables) in the section `#output`. Note that the lines of this section do not contain equations, but algebraic expressions. The time courses of all outputs and all variables defined in the section `#layer connections` are automatically visualized when running the C MEX, MATLAB or *Mathematica* model files.
The lines of the section `#output` are the legends of the plots when executing the model files. If a more descriptive legend is preferred, new variables can be defined in the section `#algebraic relations` (e.g. `Out1=...`). These variables then may be defined as outputs in the section `#output` (e.g. only `Out1` within one line). This leads to the name of the defined output variable (e.g. *Out*1) being the legend of the corresponding plot.
The automatic addition of all variables defined in the section `#layer connections` to the output list of the *Mathematica*, C MEX and MATLAB models is performed as they correspond to macroscopic quantities that are often of interest. It can be disabled by the command line option `OutLC=0`. In the SBML model, each line of the section `#output` is interpreted as a SBML 'speciesConcentrationRule'. The species notation there is: 'Output' followed by a counter starting from zero.

3 Order of assignments

3.1 Order of sections

The order of the sections in the model definition is not of interest. The sections where algebraic assignments can be defined are evaluated in the following order: `#parameters`, `#clamped concentrations`, `#molecular balances`, `#layer connections`, `#algebraic relations`. This means that e.g. all variables whose values are defined in the other sections can be used in the section `#algebraic relations`. Variables defined in the section `#algebraic relations`, however, cannot be used in all the other sections (but of course in the sec-

tions `#output` and `#reactions`). An exception is the *Mathematica* model, where the order is irrelevant.
In principle it is possible to perform definitions in other sections than the one that is intended for this. As an example, the clamping of concentrations and the definition of conservation relations or binding partners ($x_i - x_i b$) could also be done in the section `#algebraic relations`. The section `#molecular balances` could also be used to clamp concentrations of defined species. However, when using the C MEX, MATLAB or SBML models, errors may occur that result from the order of the sections. When using a section for assignments that should be done in other sections, it is possible that the so defined variables are required in other assignments or macrostates before they are defined. This problem (and other problems) can be avoided by performing every definition in the corresponding special section.

3.2 Order within sections

When using variables that are defined within the same section, they have to be defined in a row that is above their first usage. The sole exception is the section `#parameters` where this strict rule is slightly relaxed. All parameter assignments where other parameters occur are moved to the end of the parameter list. This is done in the order they are defined. All other parameter assignments are sorted alphanumerically and placed above.

4 Syntax restrictions

4.1 Tabs and spaces

Space characters are automatically removed from the model definition. Therefore, space characters are allowed at any desired position within the sections of the model definition.
In the section `#reactions`, tabs are necessary syntax elements between the reaction equation and the forward parameter and between the forward and backward parameters. Additional tabs in the section `#reactions` are allowed before reaction symbols (to align reactions) and comments and at the beginning of each line. In all other sections, tabs are automatically removed and therefore allowed at any desired position.

4.2 Beginning of lines and comments

After potential leading tabs and spaces, lines within sections have to start with a number, a letter or a reaction symbol ('->','<-' or '<->'). Otherwise they are ignored. A warning is given if the line does not start with one of the comment symbols '#' or '%' and is not empty. Comment symbols may also be used within the sections to define the rest of the line as a comment. Outside the sections, special comment symbols are not needed to place comments.
Note that rows intended to be a comment but starting with '#name' are interpreted as start of the section `#name` if this section exists in ALC. Using '%' to indicate comments avoids this problem.

4.3 Species and other variables

Syntax restrictions on molecule definitions, species, parameters and other variables are summarized in Tables 1 and 2. In general, only letters and numbers are allowed for the notation of variables. Squared and curly brackets can be used in the special contexts of species notations and molecule definitions, respectively. For species, underscores are also allowed.
Molecule definitions start with the molecule name (starting with an uppercase letter). The molecule name can be followed by a sequence of site definitions. A site definition consists of all possible configurations of this site that are separated by commas and encapsulated by curly brackets.
The characters (uppercase) 'X' and 'P' as site modifications have predefined meanings. The modification 'X' at a certain site indicates a macrostate or a pattern and therefore is not allowed in the molecule definitions. The modification 'P' indicates the phosphorylation of a binding site to which effector binding is described in another layer.
The notations of species and macrostates start with the molecule name as defined in the section `#molecules`. If there are defined sites, the total configuration is encapsulated by squared brackets, where the modifications of the distinct sites are separated by commas.
When working with the C MEX, MATLAB or SBML model you should know that the symbols '[',']' and ',' are not allowed there for the notation of variables. This problem is automatically handled by ALC. During the generation of the C MEX, MATLAB and SBML model files, ']' is removed, while '[' and ',' are replaced by underscores. This can lead to the problem that the notations of distinct species (e.g. R[0,0] and R_0_0) are identical in the C MEX, MATLAB and SBML models. An error message is given in such cases.

4.4 Complexes

The notation of complexes is according to the simplified notation that is introduced in the manuscript and in [1]. Complexes of species are treated as species of one of the corresponding molecule definitions or as species of a new molecule definition. An example for the definition of such a new molecule is the dimerization or association of two molecules as shown in chapter 2.2.2. As another example, the molecule definition of the complex of `R{0,L}{0,P}` and `R1{0,A}{0,B}` could simply be `R2{0,L}{0,P}{0,A}{0,B}`. Note that in this case the binding sites for complex formation

are not explicitly defined. This is implicitly done by the molecule definitions, where both proteins and the complex are defined as separate molecules.

Binding of a molecule with just one site, e.g. L, to a scaffold , e.g. R , is not problematic, as the complex of $R[0,0]$ and L could be $R[L,0]$. Note that in this case the binding site on L is not explicitly defined as a site. Many associations of this type in the same layer can easily be realized.

Complexes of more species with many sites can also be handled, but occur mainly in conventional mechanistic modeling. They are very atypical in layer-based modeling, as the presence of all-or-none interactions usually leads to the division of binding processes on separate layers. The handling of complexes of more than two species is demonstrated on a small example system. The scaffold A can bind B and $B1$, B can bind C, $B1$ can bind $C1$. The two binding sites on A do not interact.

```
#molecules
A{0,B,BC}{0,B1,B1C1}
B{0,C}
B1{0,C1}
C
C1
#end molecules

#reactions
A[0,X]+B[0]<->A[B,X]   k1   k1d
A[0,X]+B[C]<->A[BC,X]   k2   k2d
A[B,X]+C<->A[BC,X]   k3   k3d

A[X,0]+B1[0]<->A[X,B1]   k4   k4d
A[X,0]+B1[C1]<->A[X,B1C1]   k5   k5d
A[X,B1]+C1<->A[X,B1C1]   k6   k6d

B[0]+C<->B[C]   k7   k7d
B1[0]+C1<->B1[C1]   k8   k8d
#end reactions
```

This model definition describes the formation of all possible complexes. Observe that all complexes are handled as species of one of the participating molecules. $A[BC,B1C1]$ for example is the complex of all five proteins, where A binds B and $B1$ while B binds C and $B1$ binds $C1$.

Using the section `#remove` (see chapter 2.2.5) and choosing the option `StrictRS=0`, this system can be modeled in an easier way if the bindings of C and $C1$ do not influence the bindings of B and $B1$ to A.

```
#molecules
A{0,B,BC}{0,B1,B1C1}
B{0,B,BC}
B1{0,B1,B1C1}
C
C1
#end molecules


#reactions
B[X]+A[0,X]<->A[X,X]   k1   k1d
A[B,X]+C<->A[BC,X]   k3   k3d

A[X,0]+B1[X]<->A[X,X]   k4   k4d
A[X,B1]+C1<->A[X,B1C1]   k6   k6d

B[B]+C<->B[BC]   k7   k7d
B1[B1]+C1<->B1[B1C1]   k8   k8d
#end reactions

#remove
B[0]
B1[0]
#end remove
```

In this model definition, the species $B[B]$ and $B1[B1]$ correspond to the species $B[0]$ and $B1[0]$ in the model definition above. The other species of B and $B1$ are also defined slightly differently as in the first model definition. Defining the molecules B and $B1$ in this way and removing the species $B[0]$ and $B1[0]$ as well as their reactions allows to generate a description of the system with a lower number of rules.

4.5 Layer connections

Notations of the sums of species with phosphorylated binding sites x_i have to start with a lowercase 'x' and are optionally followed by a sequence of letters and digits. The last symbol of the notation of x_i may not be 'b'. The sums of species with occupied binding sites x_ib have the same notation as the corresponding x_i, followed by a lowercase 'b'.
Species representing the sums of molecular species with unoccupied phosphorylated binding sites act as binding partners. They have to be defined as differences of x_i and x_ib. The notation of these species has to match the syntax restrictions on species notation. The first symbol of the notation is a capital letter, optionally followed by an arbitrary sequence of alphanumeric symbols (letters, digits and the underscore character). Comma-separated sequences of alphanumeric symbols encapsulated by one pair of squared brackets can be optionally appended. See Table 1 for details about the notation of x_i, x_ib and the binding partners.

4.6 Parameters

Parameter notations start with a lowercase letter (which may not be 'r' or 'x' as this may clash with the definitions of reaction rates or layer connections) and are optionally followed by a sequence of letters and digits (see Table 1).

When working with *Mathematica*, we recommend not to denote any parameter as c. The concentration of the species $R[0,0]$ for example is handled as $c[''R[0,0]'']$ in the *Mathematica* model. Using c may therefore cause problems. You may use e.g. $c1$ instead.

4.7 Numerical values

The format of numerical values in ALC is relatively flexible. Numerical values can be given in decimal notation (e.g. 0.0023) or scientific notation (e.g. 2.3*10^(-3)). An alternative format (e.g 2.3e-3) is also allowed, but only for initial conditions, parameters and the command line options `SimTime`, `InCond` and `Param`. Note that the first of the scientific notation formats (2.3*10^(-3)) is supported by *Mathematica* and MATLAB, while the other one (2.3e-3) is supported by C MEX, MATLAB and SBML. For the generation of model files, ALC converts numerical values into the right format.

4.8 Stoichiometric coefficients and multiplication

The multiplication symbol within assignments is always '*', whereas stoichiometric coefficients in reaction equations are not followed by '*'. As an example, '2R[0,0]' has to be used within the chemical reaction equations (however not within the parameter part of reactions), everywhere else '2*R[0,0]' has to be used instead.

4.9 Compartments

Compartments are not explicitly supported by ALC. However, there are two possibilities to realize compartments with different volumes and transport between them in a model. We exemplify this on a molecule `A{Comp1,Comp2}` that is transported between compartment 1 with volume $v1$ and compartment 2 with volume $v2$.

The first possibility is to balance amounts of substances. In this case, $A[Comp1]$ is the amount of substance (not the concentration) of the species $A[Comp1]$. The transport can easily be realized as the reaction

```
A[Comp1]<->A[Comp2]   k1   k2
```

results in the model equations below.

$$r_0 = k1 * A[Comp1] - k2 * A[Comp2]$$

$$\frac{d}{dt}A[Comp1] = -r_0$$
$$\frac{d}{dt}A[Comp2] = r_0$$

The second possibility is to balance concentrations and to split the reactions for the transport between the compartments. In the first step, the reversible transport reaction is replaced by two irreversible reactions, namely the forward reaction and the backward reaction. Then each irreversible reaction is replaced by a synthesis and a degradation reaction, where the species is synthesized in one compartment and degraded in the other one. Altogether, the reversible transport of A from compartment 1 to compartment 2 can be described by the reactions

```
A[Comp1]->     k1
->A[Comp2]     k1*A[Comp1]*v1/v2
A[Comp2]->     k2
->A[Comp1]     k2*A[Comp2]*v2/v1
```

which lead to the model equations below.

$$r_0 = k1 * A[Comp1]$$
$$r_1 = k1 * A[Comp1] * v1/v2$$
$$r_2 = k2 * A[Comp2]$$
$$r_3 = k2 * A[Comp2] * v2/v1$$

$$\frac{d}{dt}A[Comp1] = -r_0 + r_3$$
$$\frac{d}{dt}A[Comp2] = r_1 - r_2$$

5 Consistency checks

Before the generation of the model files, ALC performs consistency checks on the model definition. All molecule definitions, rules, reactions, assignments and outputs are checked if they match the corresponding general format. In addition, it is verified that no variable is defined more than once. The more specific checks are listed below.

Critical inconsistencies result in error messages indicating the nature and the location of the errors. In this case, the model generation is not continued. Minor inconsistencies, e.g. undefined initial conditions or parameter values, result in warning messages. The model generation is continued and the model files will be executable. In the case of missing layer connections, the model files will often not be executable without errors. These defects in layer connections are critical errors. However, if the aim is to build only a single layer, missing layer connections can be defined in another layer and need not to be included in the model definition. Therefore, only warnings are given and the model generation is continued.

5.1 Molecule definitions

An error message is given if a site definition contains 'X' as a possible modification or if the same modification is defined twice for the same site.
An error message is also given if the notations of different species collide in the C MEX, MATLAB and SBML models. As in this formats squared brackets and commas are not allowed, ALC changes the notation of variables before writing the model files (e.g. $R[0,0]$ is replaced by R_0_0). Therefore, different species (in this case $R[0,0]$ and R_0_0) can have the same notation in the C MEX, MATLAB and SBML models.
The SBML model outputs are denoted as 'Output' followed by a counter starting from zero (e.g. $Output0$ for the first output). An error message is given if molecule definitions collide with this automatic output notation. If there are for example four outputs, species notations may not be $Output0$ to $Output3$. $Output4$ and higher, however, is allowed.

5.2 Used variables

It is checked if each used variable is according to syntax restrictions and molecule definitions. If, due to syntax restrictions, a notation of a species or a parameter is not allowed or if a used species is not defined, an error message is given. Note that species defined in the sections `#layer connections` and `#algebraic relations` do not have to be defined in the section `#molecules`.
There is a warning if a species that is balanced by an ODE shows no turnover. Note that each species of each molecule definition is balanced by an ODE, except for species whose values are defined by algebraic assignments.

5.3 Initial conditions and parameters

ALC checks if all necessary initial conditions are defined. Species whose values are defined by algebraic assignments (no matter in what section they are defined) need no initial condition. If a necessary initial condition is not set, a warning is given and the initial condition is set to the default value. The same holds true for parameter values. Additionally, there are warnings if defined parameters are not used in the model definition, and if species whose initial conditions is set are not defined by a molecule definition.

5.4 Rules and reactions

If more than one site modification of the same molecule is changed in one rule or reaction a warning is given. More than one transition to 'P' in the same reaction results in an error message. The model generation in this case is continued, however, the used correction term may be not the desired one. One of two possibilities can be chosen for constraints on rule definitions (command line option `StrictRS`, see chapter 2.2.2).

5.4.1 Minimal syntax restrictions

For `StrictRS=0` there is only one minimal requirement for the successful evaluation of rules. This is that from the left to the right on both sides of each reaction equation corresponding sites with a modification 'X' have the same site definition. If this restriction is violated, an error message is given. Overhanging sites with a modification 'X' are allowed.

5.4.2 Strict syntax restrictions

The same checks as for `StrictRS=0` and some additional checks are performed for `StrictRS=1`. For both sides of the reaction equation, one definition is constructed, which is the sequence of all molecule definitions from the left to the right where molecule names are removed. Stoichiometric coefficients are considered. Error messages are given if the positions of 'X' or the molecule definitions do not match on both sides of the reaction equation.
An overhanging end of one sequence of molecule definitions results in a warning if one of the corresponding sites has a site modification 'X' and if there is at least one site modification 'X' on both sides of the rule. This last limitation has the justification that no warnings are desired for rules that define simple degradation reactions.

5.5 Layer connections

It is checked if there is a x_ib and a variable that is a difference of x_i and x_ib for each x_i.
The definition of the difference $x_i - x_ib$ may be more complicated and include parameters. It only has to contain x_i, x_ib and a '-' sign. It is also checked if there is a x_i for each x_ib. Detailed warnings are given, if the layer connections are not defined properly.
There is a warning if the algorithm to choose the correction term c_i for a dephosphorylation reaction finds no appropriate c_i.

6 Installation

ALC can be used offline and online via a form on the ALC website (http://layer.mpi-magdeburg.mpg.de).

Offline use: The offline installation of ALC consists in extracting the file 'download_ALC.zip' in the directory where later on the model definition files will be stored. The file 'download_ALC.zip' is the recent release of ALC that can be downloaded from the ALC website. The first release of ALC is given as an Additional file of the manuscript presenting ALC.

ALC only utilizes functionalities provided in the standard Perl distribution (5.6.1 or later). Therefore, no additional modules are necessary and ALC can be directly used if Perl (www.cpan.org) is installed.

Online use: ALC can also be accessed using a browser without any additional software. Accessing ALC via the form on the ALC website (http://layer.mpi-magdeburg.mpg.de) provides full functionality but is limited to models with no more than 500 species.

7 Running ALC

There are two possible ways of running ALC. The first is to use the form on the ALC website (http://layer.mpi-magdeburg.mpg.de).
The second possibility is to use ALC offline. Note that Perl (freely available, e.g. www.cpan.org) is required for the offline use. If ALC is installed, store the model definition in the file 'layer.alc', open a command line interface and type

`ALC.pl`
or
`perl ALC.pl`

followed by the return key to perform the generation of the model files. These commands execute ALC with default values, e.g. for undefined parameters or initial conditions and for the filenames of the model definition file and the model files. The default values can be changed in the file `Config_ALC.txt`. If this file does not exist in the present directory, the default values shown in Table 3 are taken. The default values can also be changed via the command line or in the form on the ALC website for each call of ALC separately.
Append `opts` to the call of `ALC.pl` to execute ALC with changed default values. `opts` is a sequence of assignments for the parameter default value (`Param`), the initial condition default value (`InCond`), the path and the filename of the model definition file (`Source`), the path and the start of the filename of the model files (`Target`) and the simulation time for the model files (`SimTime`). By command line options it can be decided if uncritical warnings (not defined initial conditions or parameters and balanced species without turnover, `Warn`), model equations (`Show`) or the time consumption of the modeling steps (`Time`) are displayed. Additionally, it can be decided if the variables defining the signal flow between the layers are automatically visualized in the model files (`OutLC`) and if very strict consistency checks are performed on rules (`StrictRS`). As an example, the command

```
ALC.pl InCond="10^(-3)" Param="1.1*10^3"
Source="input.alc" Target="output"
```

calls ALC and sets all undefined initial conditions to 10^{-3} and all undefined parameters to $1.1 \cdot 10^3$. In this example, the model definition is read in from the file 'input.alc' and the notation of the model files starts with 'output'. The inverted commas encapsulating the values of the options can be omitted, if there are no special characters (e.g. '^') inside.

All assignments can be omitted, in this case the corresponding default values are taken. The order of the assignments is arbitrary. For details about default values and command line parameters see Table 3. Default parameters can also be easily set in the form on the ALC website.

8 Model files

The model equations that are defined by the model definition file (default, if the command line option `Source` is not defined: layer.alc) are given in MATLAB (default, if the command line option `Target` is not defined: modelM.m and modelM_call.m), C MEX (default: modelC_call.m, modelC_mdl.mdl and modelC.c), *Mathematica* notebook (default: model.nb) and *Mathematica* input file (default: model.mma.m) formats, as directly executable files. They include the visualization of the user-defined output variables and - in the case `OutLC=1` (which is the default value) - the visualization of all variables defined in the section `#layer connections`. The model is also given in SBML (default: model.xml), LaTeX (default: model.tex) and plain text (default: model.txt) formats. Finally, the model definition is converted to the LaTeX format (default: model.input.tex).

Most models consist of one file. The exceptions are the MATLAB and C MEX models. The C MEX model consists of three files. One of them (default: modelC.c) contains the S-function which is written in C, the next (default: modelC_mdl.mdl) is a file in the model description language of Simulink that defines the communication with the S-function and is called by the third (default: modelC_call.m) which also contains the definitions of initial conditions and parameters as well as the visualization of the simulation results. This file is written in the programming language MATLAB. The (standard) MATLAB model consists of two files. One of them (default: modelM.m) contains the M-function and is called by the other one (default: modelM_call.m), which also contains the definitions of initial conditions and parameters as well as the visualization of the simulation results.

9 Frequent problems

9.1 Division by zero

Setting initial conditions to zero (e.g. by choosing `InCond=0`) may result in division by zero when simulating the model. Initial conditions have to guarantee $x_i > 0 \; \forall \; i$ to avoid this problem.

9.2 Negative concentrations

Another problem that can arise during the assignment of initial conditions is that the concentrations of binding partners ($x_i - x_i b$) can be negative. Initial conditions have to guarantee $x_i \geq x_i b \; \forall \; i$ to avoid this problem.

Negative effector concentrations can also occur if the species providing the binding site in another layer rapidly 'vanish' due to dimerization. Defining a common x_i for the binding sites of the monomer and the dimer solves this problem.

Another source of negative concentrations are conservation relations. If the sum of all assigned initial conditions is larger than the total concentration of the protein, the concentration of the species that is defined by the conservation relation is negative.

9.3 Command line

The error message 'ALC.pl: No match.' (Linux) or 'error: option ... not defined' (Windows) occurs if values for command line options contain special characters and are not encapsulated by inverted commas (see Table 3).

9.4 MATLAB and C MEX

'??? Undefined function or variable "name".': The consistency checks of ALC are quite strict, so the normal case is that this error message occurs though the variable *name* is defined. In this case, *name* is used before it is defined. See chapter 3 for details about the order of assignments.

9.5 C MEX

Potentiation: In the C MEX format, potentiation using '^' is not defined. ALC converts expressions as `(...)^(...)` with or without brackets (e.g. `k1^3` or `(a+R[0,0])^(k1+3*10^2)`) into the right format (using the C-function `pow()`).

Note that consecutive and nested application of '^', e.g. `2^k1^3` or `2^(3+k1^4)` does not work when using a C MEX model created by ALC. Nested and consecutive application of potentiation can often be avoided by applying basic arithmetics. When working with numerical values, one nested potentiation using the basis ten and integer (positive or negative) exponents as in chapter 4.7 is allowed (e.g. `2^(2.5*10^3)`).

'S-function parameter count mismatch.': This error message occurs if a new C MEX model with the same file names but a different number of parameters is executed. Restarting MATLAB solves the problem.

MATLAB version: The earliest MATLAB version tested that works with the C MEX models is 7.0.4.352.

9.6 *Mathematica*

How to run the *Mathematica* input file: Input files are executed using the 'Get' command from a *Mathematica* notebook, e.g. by `<< "model.mma.m"`. If *Mathematica* was not started from the directory where the file 'model.mma.m' is located, a relative path has to be given.

9.7 SBML

Algebraic assignments: Though algebraic assignments are allowed by the SBML standard [6], not all SBML-compatible tools support them. Therefore, the SBML model files can not be used with all tools.

MathSBML: MathSBML [7] supports algebraic assignments. However, there are problems with parameter values that have an exponent with an absolute of seven or larger. In this case an error message 'Nonatomic expression expected at position 1..' can occur. This problem should not occur when using MathSBML release 2.7.0.3 or higher.

References

[1] Koschorreck M, Conzelmann H, Ebert S, Ederer M, Gilles ED: **Reduced modeling of signal transduction - a modular approach.** *BMC Bioinformatics* 2007, **8**:336.

[2] Blinov ML, Faeder JR, Goldstein B, Hlavacek WS: **BioNetGen: software for rule-based modeling of signal transduction based on the interactions of molecular domains.** *Bioinformatics* 2004, **20**(17):3289–91.

[3] Faeder JR, Blinov ML, Goldstein B, Hlavacek WS: **Rule-based modeling of biochemical networks.** *Complexity* 2005, **10**:22–41.

[4] Hlavacek WS, Faeder JR, Blinov ML, Posner RG, Hucka M, Fontana W: **Rules for modeling signal-transduction systems.** *Sci STKE* 2006, **2006**(344):re6.

[5] Blinov ML, Yang J, Faeder JR, Hlavacek WS: **Graph theory for rule-based modeling of biochemical networks**. *Lect. Notes Comput. Sci.* 2006, **4230**:89–106.

[6] Hucka M, Finney A, Sauro H, Bolouri H, Doyle J, Kitano H, Arkin A, Bornstein B, Bray D, Cornish-Bowden A, Cuellar A, Dronov S, Gilles E, Ginkel M, Gor V, Goryanin I, Hedley W, Hodgman T, Hofmeyr JH, Hunter P, Juty N, Kasberger J, Kremling A, Kummer U, Novère NL, Loew L, Lucio D, Mendes P, Minch E, Mjolsness E, Nakayama Y, Nelson M, Nielsen P, Sakurada T, Schaff J, Shapiro B, Shimizu T, Spence H, Stelling J, Takahashi K, Tomita M, Wagner J, Wang J, Forum S: **The systems biology markup language (SBML): a medium for representation and exchange of biochemical network models.** *Bioinformatics* 2003, **19**(4):524–31.

[7] Shapiro BE, Hucka M, Finney A, Doyle J: **MathSBML: a package for manipulating SBML-based biological models.** *Bioinformatics* 2004, **20**(16):2829–31.

Tables

Table 1: Syntax restrictions for the notation of entities

Note that within molecule definitions, 'X' as a site modification is not allowed. This site modification is reserved to indicate macrostates and patterns. The notations of x_i are not allowed to end with 'b', as this is reserved for the notations of x_ib. Alphanumeric symbols in this contribution are letters, digits and the underscore character.

Entity	Name	Subsequent definition	Examples
Molecule definition	Uppercase letter, then optional alphanumeric symbols	Optional: sequences of comma-separated combinations of alphanumeric symbols encapsulated by curly brackets	`R{0,P}{0,p}` `Ab1{0,L1d}` `B{1}, C`
Species or macrostate	Uppercase letter, then optional alphanumeric symbols	Optional: sequence of comma-separated combinations of alphanumeric symbols encapsulated by one pair of squared brackets	$R[0,p]$ $Ab1[L1d]$ $B[1]$, C
Parameter	Lowercase letter, not 'x' or 'r'	Optional: sequence of letters and digits	$k1$, $a34$ f, $g5b$
Layer Connection: x_i	Lowercase 'x'	Optional: sequence of letters and digits	x, $x1A$ $xB4$
Layer Connection: x_ib	Name plus subsequent definition of x_i	Appended 'b'	xb, $x1Ab$ $xB4b$

Table 2: Regular expressions for entities

Note that within molecule definitions 'X' as a site modification is not allowed. This site modification is reserved to indicate macrostates and patterns. The regular expression allows 'X', however, its occurrence is checked during the consistency checks of the model. Notations of x_i may not end with 'b' as this is reserved for the notations of x_ib. The regular expression allows the notation of x_i to end with 'b', however, internally a final 'b' indicates x_ib. Alphanumeric symbols in this contribution are letters, digits and the underscore character. A less condensed definition of syntax restrictions is given in Table 1.

Entity	Perl regular expression
Molecule definition	`[A-Z]\w*(?:\{\w+(?:,\w+)*\})*`
Species or macrostate	`[A-Z]\w*(?:\[\w+(?:,\w+)*\])?`
Parameter	`[a-qs-wyz][a-zA-Z\d]*`
Layer connections: x_i and x_ib	`x[a-zA-Z\d]*`

Table 3: Command line parameters and default values

For running ALC with changed default values type for example:

`ALC.pl InCond="2.1*10^(-2)" Source="in.alc" Target="out" Show="1" SimTime="1.5"`

The order of the options is arbitrary, the default value is taken if an option is omitted. The default values are defined in the file `Config_ALC.txt`. If this file does not exist, the values in this table are taken. The inverted commas encapsulating the value of the options can be omitted if there are no special characters inside. Default values can also be changed in the form on the ALC website.

Option	Description	Default value	Example
`Source`	Name of the model definition file[1]	layer.alc	`Source=input.alc`
`InCond`	Value for undefined initial conditions[2]	0.001	`InCond="3.1*10^2"`
`Param`	Value for undefined parameters	1	`Param="2.5*10^3"`
`SimTime`	Simulation time for the model files	100	`SimTime=10`
`Target`	Start of the names of the model files[3]	model	`Target=output`
`OutLC`	Add (1) or do not add (0) variables defined in the section `#layer connections` to the output list	1	`OutLC=0`
`Warn`	Show (1) or do not show (0) warnings for undefined initial conditions and parameters and for balanced species without turnover	1	`Warn=0`
`Show`	Show (1) or do not show (0) model equations	0	`Show=1`
`Time`	Show (1) or do not show (0) the time consumption of the modeling steps	0	`Time=1`
`StrictRS`	Perform (1) or do not perform (0) very strict consistency checks on rules[4]	1	`StrictRS=0`

[1] A relative path can also be given. As an example, `Source="test/layer.alc"` or `Source="test\layer.alc"` directs ALC to use 'layer.alc' in the directory 'test' as the model definition file. Use your system specific notation for the relative path[5].

[2] Setting `InCond=0` may result in division by zero when simulating the model. Initial conditions have to guarantee $x_i > 0 \; \forall \; i$ to avoid this problem. Initial conditions also have to guarantee $x_i \geq x_i b \; \forall \; i$ to prevent negative concentrations of binding partners.

[3] A relative path can also be given, e.g. `Target="test/model"` or `Target="test\model"` where 'test' is a directory and 'model' is the start of the name of the model files. Use your system specific notation for the relative path[5]. The directories in the path have to exist, ALC will not create them.

[4] `StrictRS=1` minimizes the probability of making unintended errors in the model definition. For `StrictRS=0` only the minimal syntax requirements that are necessary to successfully finish the modeling procedure are checked.

[5] As there are sometimes strange paths and directory names, assigning the relative path will not always work if the filename or the name of the directories include special characters. If the actual directory is desired as the relative path, give the filename (`Source`) or the start of the filenames (`Target`) directly.
