# Supplementary material for: ALC: automated reduction of rule-based models
Source: BMC Syst Biol. 2008 Oct 31;2:91. doi: 10.1186/1752-0509-2-91 (PMC2636783; doi:10.1186/1752-0509-2-91)
Supplement: Additional file 4 — ALC tutorial. The ALC tutorial extensively describes the modeling of an example system. [file 1752-0509-2-91-S4.pdf]

Automated Layer Construction (ALC) - Tutorial

Markus Koschorreck and Ernst Dieter Gilles

Release 1.0, October 2008

1 Introduction

In this tutorial, we exemplify automated modeling for a small example system. Assume a receptor R that can bind the ligand L. We consider one additional binding site which undergoes autophosphorylation and is influenced by the state of the ligand binding site. Therefore, a graded interaction [1] is present between the processes of ligand binding and autophosphorylation. An effector E can bind to the phosphorylated binding site on the receptor R which leads to a receptor-effector complex RXE. For steric reasons, the binding of E protects the binding site on R from dephosphorylation, while E can only bind if the binding site is phosphorylated. This means that an all-or-none interaction [1] is present between the processes of binding site phosphorylation and effector binding. The phosphorylated receptor can phosphorylate E that is bound to other receptors. Free E can only be dephosphorylated. Therefore, a graded interaction is present between the processes of effector binding and effector phosphorylation. Free and unphosphorylated E is degraded and synthesized. We do not consider synthesis or degradation of the receptor. Note that this example system is very similar to the example system in the manuscript and additionally includes phosphorylation of the effector, as well as synthesis and degradation of free unphosphorylated E. Layer-based modularization, according to the interaction graph of the ligand binding, receptor phosphorylation, effector binding and effector phosphorylation processes is shown in Figure 1.

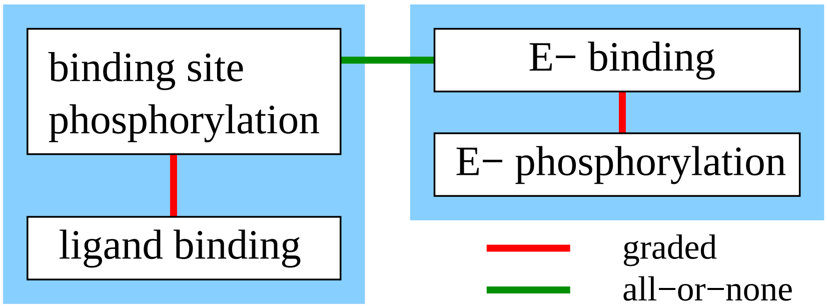

Figure 1: Interaction graph: processes, interactions and layers
Processes are shown in white boxes. The interactions between the processes are indicated by green (all-or-none interactions) and red (graded interactions) lines. The two layers are indicated by blue boxes.

2 The model definition

The model definition is divided into sections. Every line between the commands `#name` and `#end name` belongs to the section 'name'. Each section can be split into an arbitrary number of blocks that are encapsulated by `#name` and `#end name`. This means in particular that distinct layers can be modeled separately.

Comments can be added anywhere in the model definition. The symbols '#' and '%' label the rest of the line as a comment. Note that lines intended to be a comment but starting with '#name' are interpreted as the start of the section `#name` if this section exists in ALC. Using '%' as the comment symbol avoids this problem. The generation of a model definition is demonstrated for the example system introduced above.

2.1 Molecule definitions

The molecules of each layer are defined separately. The molecule definitions of the receptor layer are:

```
#molecules
R{0,L}{0,P}
L
#end molecules
```

Those of the effector layer are:

```
#molecules
E{0,p}
RXE{0,p}
#end molecules
```

In each line the name of a molecule is given, followed by the definitions of all the sites of this molecule. Molecule notations start with an uppercase letter, a site definition consists of the comma-separated sequence of all possible modifications within curly brackets.

As an example, `R{0,L}{0,P}` means that R has two sites. The first can be in the states '0' and 'L', while the second can be in the states '0' and 'P'. As an example for species notation, the receptor species with L bound to the first site and phosphorylated second site is $R[L, P]$.

According to the layer based approach [1], (uppercase) 'P' is a reserved site modification and indicates that the site is a binding site to which the effector binds in another layer. This site modification indicates phosphorylation without distinguishing if an effector is bound to this site or not. General syntax restrictions on molecule definitions, as well as on the notation of species can be found in Table 1.

Note that E and RXE have the same site definitions, as RXE is receptor bound E. According to the interaction graph (Figure 1), modifications of the receptor are not considered in the layer describing the binding of E to the receptor. Instead of defining E and RXE as separate molecules, it is also possible to define a single molecule with one additional site that indicates binding to the receptor, e.g. `E{0,R}{0,p}`.

2.2 Rules and reactions

Rules [2] and reactions are defined in the section `#reactions`. The reaction symbol is '<->' for reversible reactions and '->' or '<-' for irreversible reactions. After the chemical reaction equation, there is at least one tab followed by the parameter of the forward reaction, followed by at least one tab and the parameter of the backward reaction. Note that no matter which reaction symbol is chosen, the forward reaction proceeds from the left hand side to the right hand side of the reaction equation. For irreversible reactions, the unnecessary parameter can be omitted. One side of the reaction equation may be empty, this allows for the realization of the synthesis and degradation of species. The reactions of the receptor layer are:

```
#reactions
L+R[0,X]<->R[L,X] k1 k1d
R[0,0]<->R[0,P] k2 k2d
R[L,0]<->R[L,P] k3 k3d
#end reactions
```

The reactions of the effector layer are:

```
#reactions
RXp+E[X]<->RXE[X] k4 k4d
RXE[0]<->RXE[p] k5*Ractive k5d
E[p]->E[0] k6
E[0]<-> k7 k7d
#end reactions
```

The hallmark of macrostates is that they must have at least one site modification 'X'. Note that e.g. RXp and $RXE[0]$ are not macrostates, as in both cases the letter 'X' is part of the molecule name and not a site modification. Macrostates usually correspond to sums of species. $E[X]$ for example, represents the sum of all unbound effector species ($E[X] = E[0] + E[p]$). A macrostate within the chemical reaction equation (not within the parameter part of the reaction) is interpreted as a pattern and marks the reaction as a rule. The first reaction of the receptor layer is a rule and indicates that both $R[0, 0]$ and $R[0, P]$ bind L with the same kinetic constants. The first reaction of the effector layer is also a rule and indicates that the binding of E to RXp is independent of the phosphorylation state of E.

As it can be seen in the second reaction of the effector layer, a reaction parameter is not necessarily just a parameter value such as $k1$. Reactions can be parameterized by arbitrary algebraic expressions that can also include species and macrostates. In the parameter part of reactions and, except for the reaction equations, everywhere else macrostates are interpreted as the sum they define.

ALC evaluates the rules according to the molecule definitions. The reactions of the receptor layer are:

$$
\begin{aligned}
L + R[0, 0] &\leftrightharpoons R[L, 0] \quad k1 \quad k1d \\
L + R[0, P] &\leftrightharpoons R[L, P] \quad k1 \quad k1d \\
R[0, 0] &\leftrightharpoons R[0, P] \quad k2 \quad k2d \\
R[L, 0] &\leftrightharpoons R[L, P] \quad k3 \quad k3d.
\end{aligned}
$$

The reactions of the effector layer are:

$$
\begin{aligned}
RXp + E[0] &\leftrightharpoons RXE[0] \quad k4 \quad k4d \\
RXp + E[p] &\leftrightharpoons RXE[p] \quad k4 \quad k4d \\
RXE[0] &\leftrightharpoons RXE[p] \quad k5 * Ractive \quad k5d \\
E[p] &\rightarrow E[0] \quad k6 \quad 0 \\
E[0] &\leftrightharpoons \quad k7 \quad k7d.
\end{aligned}
$$

2.3 Algebraic relations

Arbitrary explicit algebraic assignments can be defined in the section `#algebraic relations`. The right hand side of these assignments may contain parameters, species and macrostates. In this case, we require an additional information transfer between the layers.

```
#algebraic relations
Ractive=R[X,P]
#end algebraic relations
```

In the assignment above, $R[X,P]$ is a macrostate and represents the sum of all phosphorylated receptor species ($R[X,P] = R[0,P] + R[L,P]$).
The notation of variables defined in the section `#algebraic relations` has to match the general restrictions on species notations (Table 1).
Note that one could also define the reaction where *Ractive* is used as

```
RXE[0]<->RXE[p]   k5*R[X,P]   k5d
```

instead of

```
RXE[0]<->RXE[p]   k5*Ractive   k5d
```

to get the same result.

2.4 Layer connections

The signal flow between the layers is defined in the section `#layer connections`. In the receptor layer, x is defined as the sum of receptor species with a phosphorylated binding site.

```
#layer connections
x=R[X,P]
#end layer connections
```

In the effector layer, xb is defined as the sum of species with an occupied binding site. RXp, a species representing the difference of x and xb (which is the sum of molecular species with an unoccupied phosphorylated binding site), acts as a binding partner for effector binding.

```
#layer connections
xb=RXE[X]
RXp=x-xb
#end layer connections
```

A special nomenclature has to be regarded (Table 1). All notations of the sums of species with phosphorylated binding sites x_i start with a lowercase 'x', the corresponding sums of species with occupied binding sites x_ib have the same notation, followed by an additional 'b'. For this reason notations of x_i are not allowed to end with 'b'. The notation of the binding partners representing their difference (which is the sum of all molecular species with unoccupied phosphorylated binding sites) only has to match the general restrictions on species notations (Table 1). Note that the binding partner has to be defined below x_i and x_ib.

2.5 Reaction rates

The reactions (section 2.2) are used to generate the reaction rates following a generalized law of mass action. The reaction rates for the receptor layer are:

$$\begin{aligned}
r_0 &= k1 * L * R[0,0] - k1d * R[L,0] \\
r_1 &= k1 * L * R[0,P] - k1d * R[L,P] \\
r_2 &= k2 * R[0,0] - k2d * ((x - xb)/x) * R[0,P] \\
r_3 &= k3 * R[L,0] - k3d * ((x - xb)/x) * R[L,P].
\end{aligned}$$

The reaction rates for the effector layer are:

$$\begin{aligned}
r_4 &= k4 * RXp * E[0] - k4d * RXE[0] \\
r_5 &= k4 * RXp * E[p] - k4d * RXE[p] \\
r_6 &= k5 * Ractive * RXE[0] - k5d * RXE[p] \\
r_7 &= k6 * E[p] \\
r_8 &= k7 * E[0] - k7d.
\end{aligned}$$

For rates describing the dephosphorylation of binding sites with a site modification 'P', the layer based approach demands an additional factor $(x_i - x_ib)/x_i$ which represents the fraction of phosphorylated binding sites that are not occupied. This factor is automatically added by ALC as can be seen for the factor $(x - xb)/x$ in the rates r_2 and r_3.

2.6 Parameters and initial conditions

Reaction parameters and initial conditions are defined in the sections `#parameters` and `#initial conditions`, respectively.

```
#parameters
totR=10
k1=2
#end parameters
```

```
#initial conditions
R[0,0]=2
#end initial conditions
```

Parameter notations start with a lowercase letter, which is not allowed to be 'r' (as it is reserved for the internal notation of reaction rates) or 'x' (which is reserved for the notation of layer connections). For more details see Table 1. In this example not all parameters used are defined by assigning a value to them. The remaining parameters are set to the default value and

a warning is given. The same holds for necessary but undefined initial conditions. Species that are defined by a conservation relation (section 2.8) or an algebraic relation (section 2.3), or that are clamped (section 2.7) do not need an initial condition.
The format of numerical values in ALC is relatively flexible. In each section, numerical values can be given in decimal notation (e.g. 0.0023) or scientific notation (e.g. 2.3*10^(-3)). Another format of scientific notation (e.g. 2.3e-3) may also be used in the sections `#parameters` and `#initial conditions`.
To avoid problems when simulating the model files, initial conditions have to guarantee $x_i > 0 \ \forall \ i$ and $x_i \geq x_i b \ \forall \ i$.

2.7 Clamped concentrations

Concentrations of defined species can be easily clamped (i.e. set to a constant value) as demonstrated for L in the receptor layer.

```
#clamped concentrations
L=10
#end clamped concentrations
```

2.8 Conservation relations

Explicit conservation relations can be defined in the section `#molecular balances`. As there is no synthesis or degradation of R, the total amount of R ($totR$) is constant. Therefore, the ODE for one species (e.g. $R[L, 0]$) can be replaced by a conservation relation for R.
The defined total amount of receptor ($totR$) has to be larger than (or equal to) the sum of all initial conditions for receptor concentrations that are defined by ODEs. Otherwise the concentration of the species, which is defined by the conservation relation, will be negative.

```
#molecular balances
R[L,0]=totR-R[X,X]+R[L,0]
#end molecular balances
```

ALC supports the unusual equation format shown above, where the species on the left hand side of the equation may also occur on the right hand side, where this recurrence is automatically removed. Macrostates that contain the species whose ODE is replaced by this conservation relation can be used. This allows for very simple definitions of conservation relations.

2.9 Output variables

Outputs, in this case unphosphorylated free E and the sum of all receptor species with a bound ligand, can be defined in the section `#output`.

```
#output
R[L,X]
E[0]
#end output
```

Not only parameters, species and macrostates can be declared as outputs but so can arbitrary algebraic expressions. The outputs and (in the default case) all variables defined in the section `#layer connections` are automatically visualized when executing the C MEX, MATLAB or *Mathematica* files created by ALC.

3 The finished model definition

The complete model definition is shown below. Remember that each section of the model definition can be divided into an arbitrary number of blocks, which allows for the modular structure of the model in the model definition to be mirrored.

```
######################
##receptor layer

#molecules
R{0,L}{0,P}
L
#end molecules

#parameters
totR=10
k1=2
#end parameters

#initial conditions
R[0,0]=2
#end initial conditions

#clamped concentrations
L=10
#end clamped concentrations

#reactions
L+R[0,X]<->R[L,X] k1 k1d
R[0,0]<->R[0,P] k2 k2d
R[L,0]<->R[L,P] k3 k3d
#end reactions

#layer connections
x=R[X,P]
#end layer connections
```

```
#molecular balances
R[L,0]=totR-R[X,X]+R[L,0]
#end molecular balances

#algebraic relations
Ractive=R[X,P]
#end algebraic relations

##end receptor layer

######################
##effector layer

#molecules
E{0,p}
RXE{0,p}
#end molecules

#reactions
RXp+E[X]<->RXE[X] k4 k4d
RXE[0]<->RXE[p] k5*Ractive k5d
E[p]->E[0] k6
E[0]<-> k7 k7d
#end reactions

#layer connections
xb=RXE[X]
RXp=x-xb
#end layer connections

##end effector layer

######################

#output
R[L,X]
E[0]
#end output
```

4 Running ALC

There are two possible ways of running ALC. The first is to use the form on the ALC website (http://layer.mpi-magdeburg.mpg.de).
The second possibility is to use ALC offline. Note that Perl (freely available, e.g. www.cpan.org) is required for the offline use. If ALC is installed, store the model definition in the file 'layer.alc', open a command line interface and type

```
ALC.pl
```
or
```
perl ALC.pl
```

followed by the return key to perform the generation of the model files. These commands execute ALC with default values, e.g. for undefined parameters or initial conditions and for the filenames of the model definition file and the model files. The default values can be changed in the file `Config_ALC.txt`. If this file does not exist in the present directory, the default values shown in Table 2 are taken. The default values can also be changed via the command line or in the form on the ALC website for each call of ALC separately.

For details see Table 2 and the user guide.

5 Model files

The finished model is given in MATLAB (default: modelM.m and modelM_call.m), C MEX (default: modelC_call.m, modelC_mdl.mdl and modelC.c), *Mathematica* notebook (default: model.nb) and *Mathematica* input file (default: model.mma.m) formats, as directly executable files. The model is also given in SBML (default: model.xml), LaTeX (default: model.tex) and plain text (default: model.txt) formats. Additionally, the model definition is converted to the LaTeX format (default: model.input.tex).

The model equations generated by ALC are as follows:

clamped concentrations and molecular balances

$$L = 10$$
$$R[L,0] = totR - (R[0,0] + R[0,P] + R[L,P])$$

macrostate definitions

$$R[L,X] = R[L,0] + R[L,P]$$
$$R[X,P] = R[0,P] + R[L,P]$$
$$RXE[X] = RXE[0] + RXE[p]$$

layer connections

$$x = R[X,P]$$
$$xb = RXE[X]$$
$$RXp = x - xb$$

algebraic relations

$$Ractive = R[X,P]$$

reaction rates

$$r_0 = k1 * L * R[0,0] - k1d * R[L,0]$$
$$r_1 = k1 * L * R[0,P] - k1d * R[L,P]$$
$$r_2 = k2 * R[0,0] - k2d * ((x - xb)/x) * R[0,P]$$
$$r_3 = k3 * R[L,0] - k3d * ((x - xb)/x) * R[L,P]$$
$$r_4 = k4 * RXp * E[0] - k4d * RXE[0]$$
$$r_5 = k4 * RXp * E[p] - k4d * RXE[p]$$
$$r_6 = k5 * Ractive * RXE[0] - k5d * RXE[p]$$
$$r_7 = k6 * E[p]$$
$$r_8 = k7 * E[0] - k7d$$

differential equations

$$\frac{d}{dt}R[0,0] = -r_0 - r_2$$
$$\frac{d}{dt}R[0,P] = -r_1 + r_2$$
$$\frac{d}{dt}R[L,P] = r_1 + r_3$$
$$\frac{d}{dt}E[0] = -r_4 + r_7 - r_8$$
$$\frac{d}{dt}E[p] = -r_5 - r_7$$
$$\frac{d}{dt}RXE[0] = r_4 - r_6$$
$$\frac{d}{dt}RXE[p] = r_5 + r_6$$

The order of the sections is the same as in the C MEX, MATLAB and SBML models. To avoid cyclic dependencies, ALC automatically replaces macrostates used for the definition of conservation relations by the corresponding sums.

References

[1] Koschorreck M, Conzelmann H, Ebert S, Ederer M, Gilles ED: **Reduced modeling of signal transduction - a modular approach.** *BMC Bioinformatics* 2007, **8**:336.

[2] Hlavacek WS, Faeder JR, Blinov ML, Posner RG, Hucka M, Fontana W: **Rules for modeling signal-transduction systems.** *Sci STKE* 2006, **2006**(344):re6.

6 Tables

Table 1: Syntax restrictions for the notation of entities
Note that within molecule definitions, 'X' as a site modification is not allowed. This site modification is reserved to indicate macrostates and patterns. The notations of x_i are not allowed to end with 'b', as this is reserved for the notations of x_ib. Alphanumeric symbols in this contribution are letters, digits and the underscore character.

Entity	Name	Subsequent definition	Examples
Molecule definition	Uppercase letter, then optional alphanumeric symbols	Optional: sequences of comma-separated combinations of alphanumeric symbols encapsulated by curly brackets	`R{0,P}{0,p}` `Ab1{0,L1d}` `B{1}, C`
Species or macrostate	Uppercase letter, then optional alphanumeric symbols	Optional: sequence of comma-separated combinations of alphanumeric symbols encapsulated by one pair of squared brackets	$R[0,p]$ $Ab1[L1d]$ $B[1]$, C
Parameter	Lowercase letter, not 'x' or 'r'	Optional: sequence of letters and digits	$k1$, $a34$ f, $g5b$
Layer Connection: x_i	Lowercase 'x'	Optional: sequence of letters and digits	x, $x1A$ $xB4$
Layer Connection: x_ib	Name plus subsequent definition of x_i	Appended 'b'	xb, $x1Ab$ $xB4b$

Table 2: Command line parameters and default values

For running ALC with changed default values type for example:

`ALC.pl InCond="2.1*10^(-2)" Source="in.alc" Target="out" Show="1" SimTime="1.5"`

The order of the options is arbitrary, the default value is taken if an option is omitted. The default values are defined in the file `Config_ALC.txt`. If this file does not exist, the values in this table are taken. The inverted commas encapsulating the value of the options can be omitted if there are no special characters inside. Default values can also be changed in the form on the ALC website.

Option	Description	Default value	Example
`Source`	Name of the model definition file[1]	layer.alc	`Source=input.alc`
`InCond`	Value for undefined initial conditions[2]	0.001	`InCond="3.1*10^2"`
`Param`	Value for undefined parameters	1	`Param="2.5*10^3"`
`SimTime`	Simulation time for the model files	100	`SimTime=10`
`Target`	Start of the names of the model files[3]	model	`Target=output`
`OutLC`	Add (1) or do not add (0) variables defined in the section `#layer connections` to the output list	1	`OutLC=0`
`Warn`	Show (1) or do not show (0) warnings for undefined initial conditions and parameters and for balanced species without turnover	1	`Warn=0`
`Show`	Show (1) or do not show (0) model equations	0	`Show=1`
`Time`	Show (1) or do not show (0) the time consumption of the modeling steps	0	`Time=1`
`StrictRS`	Perform (1) or do not perform (0) very strict consistency checks on rules[4]	1	`StrictRS=0`

[1] A relative path can also be given. As an example, `Source="test/layer.alc"` or `Source="test\layer.alc"` directs ALC to use 'layer.alc' in the directory 'test' as the model definition file. Use your system specific notation for the relative path[5].

[2] Setting `InCond=0` may result in division by zero when simulating the model. Initial conditions have to guarantee $x_i > 0 \; \forall \; i$ to avoid this problem. Initial conditions also have to guarantee $x_i \geq x_i b \; \forall \; i$ to prevent negative concentrations of binding partners.

[3] A relative path can also be given, e.g. `Target="test/model"` or `Target="test\model"` where 'test' is a directory and 'model' is the start of the name of the model files. Use your system specific notation for the relative path[5]. The directories in the path have to exist, ALC will not create them.

[4] `StrictRS=1` minimizes the probability of making unintended errors in the model definition. For `StrictRS=0` only the minimal syntax requirements that are necessary to successfully finish the modeling procedure are checked.

[5] As there are sometimes strange paths and directory names, assigning the relative path will not always work if the filename or the name of the directories include special characters. If the actual directory is desired as the relative path, give the filename (`Source`) or the start of the filenames (`Target`) directly.
